# *De Novo* Design of Peptide Masks Enables Rapid Generation of Conditionally-Active Miniprotein Binders

**DOI:** 10.1021/jacs.5c16108

**Published:** 2025-11-24

**Authors:** Montserrat Escobar-Rosales, Cristina Montaner, Marc Expòsit, Roberta Lucchi, Cristina Díaz-Perlas, David Baker, Benjamí Oller-Salvia

**Affiliations:** † Institut Químic de Sarrià (IQS), 82824 Universitat Ramon Llull, Via Augusta 390, Barcelona 08017, Spain; ‡ Department of Biochemistry, 7284 University of Washington, Seattle, Washington 98195, United States; § Institute for Protein Design, University of Washington, Seattle, Washington 98195, United States; ∥ Howard Hughes Medical Institute, University of Washington, Seattle, Washington 98109, United States

## Abstract

The widespread expression of therapeutic targets in both diseased and healthy tissues poses a major challenge for protein-based therapeutics, often leading to dose-limiting side effects. One promising strategy to enhance selectivity is reversible inactivation via affinity masks tethered through cleavable linkers responsive to disease-specific cues. Here, we introduce a workflow for the *de novo* design of peptide masks that reversibly inactivate miniprotein binders. By extending the C-terminus of the binder with a protease-cleavable linker and a masking helix, we generated minimal constructs that sterically block the receptor-binding interface. We applied this strategy to four therapeutically relevant targets, EGFR domains I and III, FGFR2, and IL7Rα, demonstrating broad applicability. Nearly half of the 20 designs achieved >100-fold affinity reduction, with the most effective mask decreasing EGFR binding by over 3 orders of magnitude. Upon cleavage by tumor-associated proteases, binding was restored in 19 out of 20 cases, confirming reversibility. We further show that micromolar or weaker affinity between the binder and the isolated mask is sufficient for robust inactivation and rapid activation. Additionally, by chemically conjugating a photocleavable linker, we created a light-responsive version of the masked binder, enabling external control with comparable efficiency to protease-sensitive designs. This work establishes a generalizable, rapid, and efficient platform for designing cleavable peptide masks from scratch, paving the way for conditionally active protein therapeutics responsive to endogenous or exogenous stimuli.

## Graphical abstract

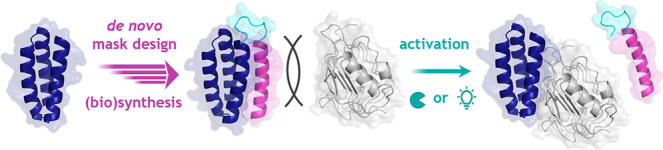

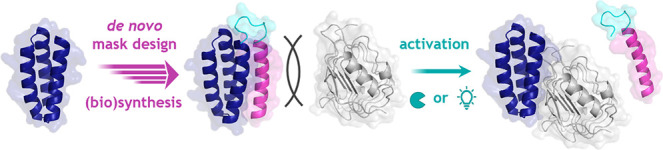

## Introduction

Protein therapeutics have transformed the treatment of diseases ranging from cancer to immune disorders, largely owing to their high target selectivity.^1^ Yet many therapeutic targets are expressed not only at diseased sites but also in healthy tissues, frequently leading to dose-limiting toxicities. A compelling strategy to improve the therapeutic index is to engineer conditionally active biologics that remain inert systemically and are activated only at the site of disease. Although multiple approaches have been explored to achieve such spatiotemporal control, their applicability varies widely across protein formats, antigen specificities, and activation stimuli.^2–4^ Expanding the toolbox of conditional activation strategies is therefore essential to enable broader therapeutic applications.

Most current methods for introducing stimulus-responsiveness into therapeutic proteins rely on a masking moiety that transiently occludes the ligand-binding interface to prevent target engagement until a cleavable linker is severed by a disease-relevant stimulus (e.g., protease activity, pH, hypoxia) or an external trigger (e.g., light, small effector molecule).^5–9^ Masks can either bind the site with defined affinity or rely on steric hindrance alone. The former generally provide higher blocking efficiency but require adaptation for each antigen specificity. A key design constraint for affinity-based masks is that they need to be strong enough to block binding when tethered, yet weak enough to dissociate rapidly once released.^10^ Cropped antigen domains including the epitope have occasionally been used as masks, but they make affinity optimization difficult, often suffer from low stability, and may demand extensive tailoring.^5,11,12^ Therefore, identifying suitable sequences has largely relied on labor-intensive display-based library screens (mostly yeast or bacterial), which are time- and resource-consuming and must be repeated for each new target.^12–18^ Here, we present the first method for the rapid *de novo* design of cleavable peptide masks that circumvents large-scale experimental screening.

Affinity-based masks have been developed for a variety of therapeutic proteins, ranging from immunoglobulins (IgGs) and derivatives such as single chain antibody variable fragments (scFvs) and antigen binding fragments (Fabs), as well as affibodies and cytokines.^2,19,20^ While all these therapeutic proteins are derived from natural scaffolds, machine learning strategies have recently revolutionized the field of protein computational modeling, enabling the generation of *de novo* designed structures. It is now possible to model proteins with near-experimental accuracy and even to design proteins with therapeutic potential from scratch.^21–23^ At least one *de novo* designed protein-based vaccine has been clinically approved, and other protein therapeutics have shown great promise in preclinical and clinical settings.^1,24^ Some designed systems have been engineered to be stimulus-responsive, such as LOCKR, a ∼40 kDa protein operator that integrates signals via displacement of a multihelix caging domain that remains attached to a helical latch; this platform has been used as a biosensor and to target cells according to predefined antigen combinations.^25^ Split *de novo* designed cytokines have also been engineered.^26^ However, the *de novo* design of cleavable affinity masks for therapeutic proteins remains an untapped field. Among *de novo* designed proteins with therapeutic potential, miniprotein binders (mbs) offer several advantages over natural biomolecule-derived binders such as immunoglobulins, including small size (down to ∼7 kDa), improved tissue diffusion, enhanced stability, high production yields, and ease of modification and characterization.^27^ An increasing number of mbs has been reported to engage a wide range of targets, many of them with high therapeutic relevance.^27^ −^28,29,30,31,32^ These binders would often also benefit from an additional layer of selectivity provided by reversible inactivation.

Masking moieties used to modulate the activity of natural binders such as antibodies and cytokines range from short peptides to single-chain antibody fragments (scFv), nanobodies, and affibodies.^14,18,20,33,34^ While antibody-derived masks are often easier to develop because their larger size provides substantial steric hindrance, minimal peptide masks offer distinct advantages: they are less likely to elicit an immune response and minimally perturb the parent binder. These properties make short peptide masks particularly suitable for small proteins, such as mbs. Designing high-affinity peptides with current computational tools is challenging and requires extensive screening. We reasoned, however, that the proximity effect conferred by covalent tethering could compensate for low intrinsic affinity, allowing short peptide masks to achieve efficient and reversible inactivation of therapeutic miniproteins.

In this work, we present a versatile and generalizable strategy for the *de novo* design of minimal peptide masks that enable reversible inactivation of mbs, bypassing labor-intensive library screening. Our approach leverages state-of-the-art machine-learning tools, including RoseTTAFold-based algorithms for backbone generation, ProteinMPNN for sequence design, and AlphaFold2 for structural validation, to create C-terminal extensions composed of a cleavable linker and a masking helix that sterically occludes the receptor-binding interface. We apply this design framework to multiple therapeutically relevant targets, demonstrating its broad applicability across mbs. The workflow incorporates *in silico* filtering to prioritize candidates with favorable structural and interface metrics, followed by experimental validation of expression, structural integrity, binding affinity, and conditional activation. We further perform an in-depth analysis of a masked mb with EGFR antagonistic activity and show that micromolar mask–binder affinity is sufficient for effective blocking and rapid activation. Beyond establishing a robust pipeline for protease-responsive masking, we also explore the adaptability of the concept to alternative stimuli, exemplified by the development of a photoactivatable variant through site-specific chemical conjugation of a light-cleavable linker.

## Results and Discussion

### *De Novo* Design of Masked Miniproteins

To demonstrate the feasibility of designing a masking peptide from scratch, we selected four *de novo* designed miniprotein binders (mbs) previously reported.^27^ We chose two mbs targeting EGFR—one for domain I and the other one for domain III—, one mb targeting FGFR2, and one against IL7Rα. These receptors are expressed at high levels on tumor or tumor-related cells but also in some healthy tissues and off-site engagement has been reported to have adverse effects.^33,35,36^ Therefore, miniproteins targeting these and other disease-related receptors would benefit from an additional layer of specificity through reversible inactivation with a mask that is selectively released at the tumor site.

Like many *de novo* designed mbs, the four selected mbs consist of three helix bundles, with two alpha helices mediating receptor interaction, while the third helix acts as a linker and a structural scaffold (Figure 1a). We hypothesized that a mask could be designed to cover the binding site between the two binding helices, thereby sterically hindering receptor engagement (Figure 1b). To create a minimal masking moiety, we envisioned adding a fourth helix linked to one terminus via a protease-cleavable sequence. In our masking design, the mask is not intended to occlude all receptor-binding residues but rather to introduce a steric barrier at the center of the interface.

**Figure 1:**
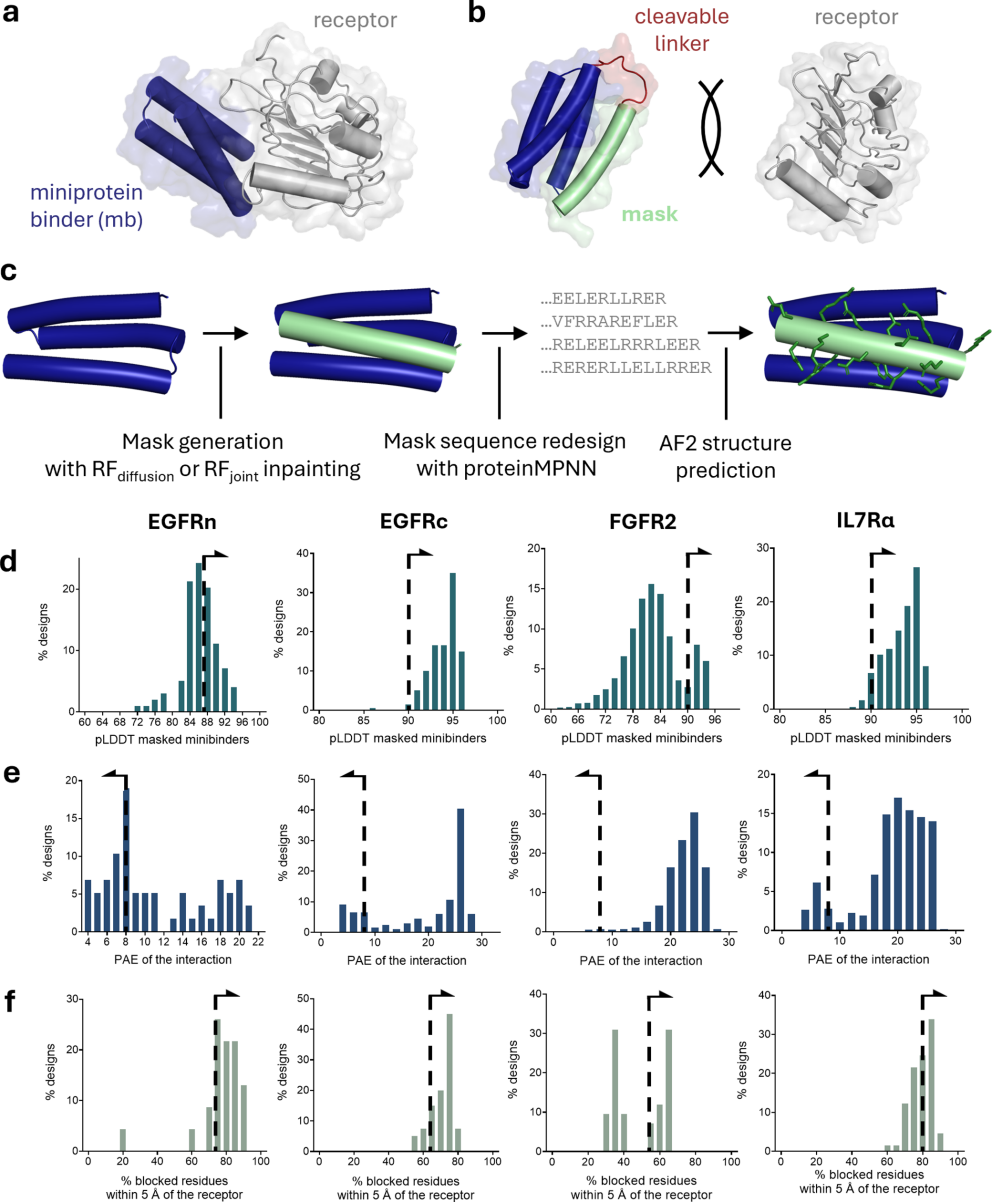
Workflow for designing masked miniproteins. (a) Complex of EGFRn_mb miniprotein (blue) and its target EGFR domain I (gray) modeled with AF2.^27^ (b) Design of an affinity mask (green) based on a C-terminal extension of the miniprotein that prevents binding to EGFR and bears a cleavable linker (red). (c) Workflow of the computational design of the mask. Residues proposed by ProteinMPNN are depicted in green. (d) Distribution of the mb pLDDT, a measure of structure confidence prediction. (e) Distribution of the mb PAE, which reflects interaction prediction accuracy. (f) Percentage of mb residues involved in the mb–receptor interaction blocked by the mask, i.e. residues in the mb at 5 Å from the receptor that are also within 5 Å of the mask.

To design a mask that fills the groove between two α-helices while preserving their conformation, we approached the mask design as a “missing information” recovery problem (Figure 1c). Thus, we generated the peptide mask utilizing RoseTTAFold diffusion (RFdiffusion) (EGFRc_mb, FGFR2_mb and IL7Rα_mb) or RF_joint_ inpainting^37^ (EGFRn_mb), both of which are machine-learning models that create viable protein backbones around given motifs. To guide the mask toward blocking the receptor-binding interface in RFdiffusion, we selected 3–4 hotspot residues at the central groove of the mb-receptor interface. Using this strategy, we generated between 100 and 1000 candidate masks per miniprotein binder.

The 15–25 residue masking sequence was then redesigned using ProteinMPNN,^38^ a deep learning model that suggests amino acid sequences for a given target structure. ProteinMPNN provided sequences for the masks that interact with the receptor-binding interface of the mb. Finally, the structures of the sequences generated were predicted with AlphaFold2 (AF2), and the models obtained were analyzed and filtered to select the best candidates for experimental validation.

We evaluated the designs based on several *in silico* criteria. For all designs, we studied the interaction between the mask and the miniprotein binder as a complex, excluding the linker. First, we selected designs with a high-confidence structure prediction, defined as complex predicted local distance difference test (pLDDT) >90 and mask pLDDT >80 (Figure 1d). Second, to ensure reliable interactions, we applied a threshold of 8 for the interchain predicted aligned error (PAE) (Figure 1e). Third, we aimed to maximize the number of receptor-interacting residues on the mb that were sterically hindered by the mask in the modeled structures, defined as residues within 5 Å of both the receptor and the mask (Figure 1f); residue coverage thresholds between 60% and 80% were used to narrow down the list to 20–40 designs per mb.

To streamline cloning and downstream analysis, we prioritized designs in which the mask was connected to the C-terminus of the miniprotein binder. Accordingly, we selected constructs exhibiting the correct orientation, i.e. those with the N-terminus of the mask positioned near the C-terminus of the mb, which represented approximately half of the total designs. From this subset, we chose 5 candidates that displayed diversity in both sequence composition and length for each mb (Figure S1). Finally, for reversible activation, we incorporated a protease-cleavable spacer between the mask and the binder including a motif recognized by matrix metalloproteinases (MMP2 and MMP9), which are overexpressed in many tumor types.^39^ Each mask was preceded by a GS linker.

Even before filtering, virtually all masks displayed an α-helical structure and most of them formed a four-helix bundle, consistent with the expected shape complementarity. Although both RF_joint_ inpainting and RFdiffusion produced viable designs, RF_joint_ inpainting yielded a higher number of masks covering the binding site. All models obtained with RF_joint_ inpainting or RFdiffusion displayed high structural similarity with the AF2 predictions, with a Cα root-mean-square deviation (rmsd) below 1.5 Å for all designs.

### Experimental Validation of Masking Efficiency and Proteolytic Activation

For each mb, the 5 selected masked designs along with the unmasked control were cloned into a pET29 vector and expressed in *E. coli* BL21­(DE3) cells under IPTG induction (Tables S1 and S2). All constructs included an N-terminal His-tag, facilitating both affinity purification and downstream detection. Purified miniproteins exhibited the expected molecular weight and high purity (>90%), as confirmed by SDS-PAGE (Figure S2) and LC–MS (Figure S3 and Table S3). Size-exclusion chromatography (SEC) analysis showed no aggregation or dimerization due to crossmasking (Figure S4). Protein yields ranged from 10 to 200 mg per liter of culture for both masked and unmasked binders, consistent with reported values for the parental mbs,^27^ indicating that the masks did not significantly alter expression.

Binding capacity was assessed on cells expressing high levels of target receptors or by ELISA. EGFRn and EGFRc binding were studied in A-431 human epidermoid carcinoma cells, while FGFR2 binding was evaluated in SK-BR-3 breast cancer cells. Incubations were performed at 4 °C to avoid internalization and unspecific cell uptake, and binding was assessed via flow cytometry. Due to low receptor expression in common cell lines, IL7Rα binding was measured using its extracellular domain by ELISA. In all cases, the IC_50_ values of the unmasked binders aligned with reported affinities: roughly 7 nM for EGFRn, 25 nM for EGFRc, 406 nM for FGFR2 and 0.4 nM for IL7Rα (Table S4), confirming proper folding and functionality.

Notably, all masked designs provided some degree of binding inhibition. Out of 20 mask designs, 9 showed over 100-fold reduction in affinity (Figures S5). This shift is comparable to state-of-the art affinity masks identified using extensive yeast and bacterial display screening campaigns^5,12,15,16,18,34^ and higher than most masking methods based on steric hindrance.^8,40–46^ Among the remaining designs, 2 showed 10–20-fold reduction, while the rest exhibited 2–5-fold shifts. EGFRn and FGFR2 masks demonstrated particularly high success rates, with over half achieving >100-fold affinity reductions (Figures 2a,c, S5a,c). For EGFRc, two masks achieved 10–100-fold reductions (Figures 2b and S5b), and for IL7Rα, two designs reduced affinity by ∼10-fold (Figures 2d and S5d). While Rosetta interface metrics (e.g., ΔΔ*G*, target-aligned RMSDs, contact molecular surface, and hydrophobic residue contacts) were consistently high across selected models, we observed no clear correlation between these parameters or the AF2 confidence scores used for filtering and the actual blocking efficiency of the masks (Figure S6). These results emphasize that, although AF or Rosetta metrics cannot yet reliably discriminate among the best-performing candidates,^47^ they are highly effective for narrowing the design space.

**Figure 2:**
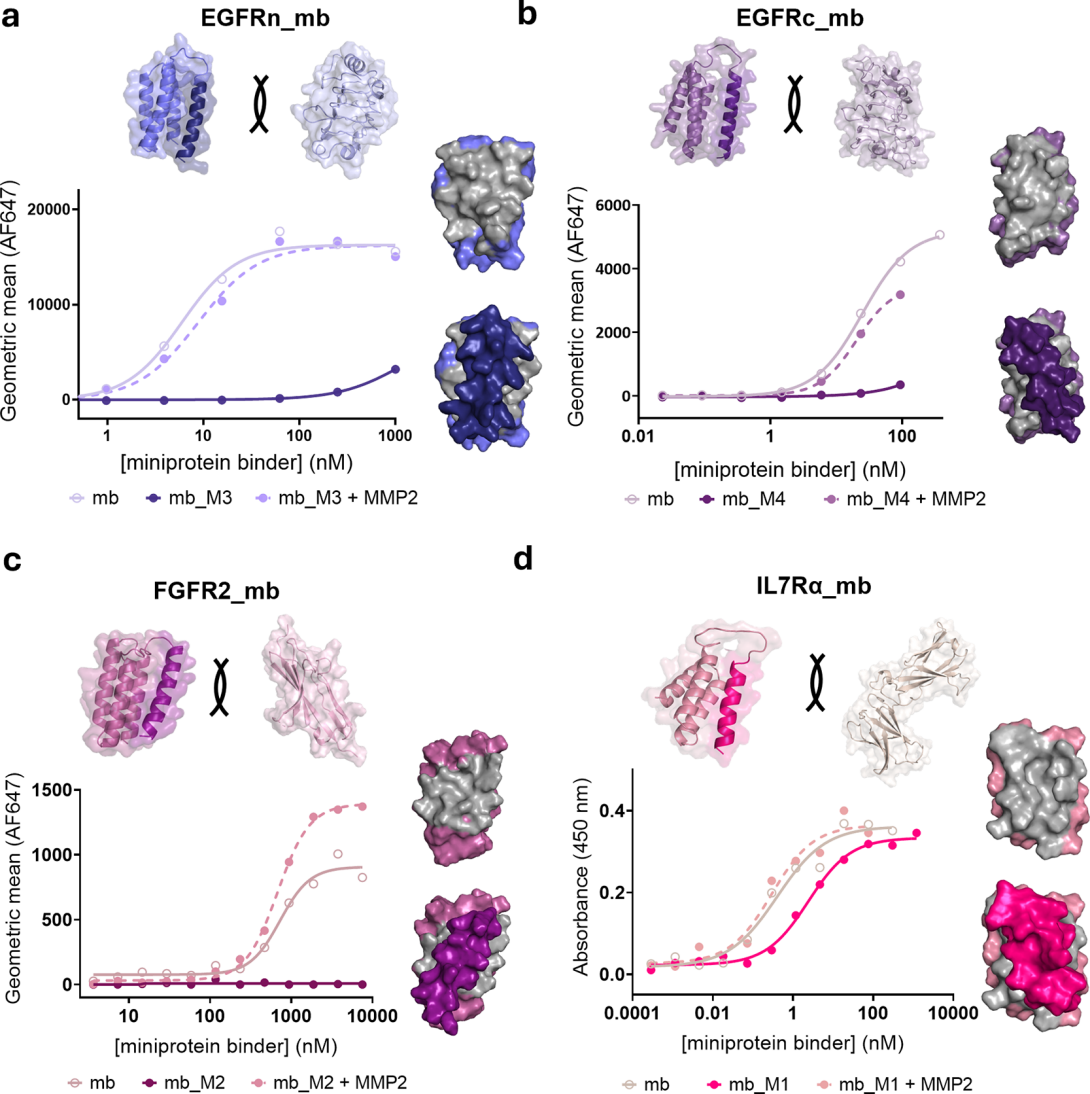
Masked miniproteins exhibit strong affinity reduction and full recovery upon protease cleavage. Selected masked miniprotein binders are shown without the mask (mb), with the mask (Mx) and with the mask after cleavage with MMP2 (mb_Mx + MMP2). The masked and unmasked mb surfaces are represented showing that the mask covers the mb-receptor interface (gray). (a) EGFRn_mb_M3. (b) EGFRc_mb_M4. (c) FGFR2_mb_M2. (d) IL7Rα_mb_M1.

The functional performance of all selected masks is remarkable given their short length (15–25 residues), a range where computational design typically faces significant challenges. This success is even more notable considering that half of the masks were designed on AlphaFold2 mb models (EGFRn and EGFRc) rather than experimentally determined structures.

To evaluate conditional activation, masked miniproteins were incubated with preactivated MMP2 for 2 h at 37 °C to ensure cleavage in all cases,^41^ although complete hydrolysis was achieved within 15 min under the tested conditions (Figure S7). Linker scission was verified by LC–MS (Figure S8). All designs were efficiently cleaved, indicating accessibility of the protease substrate regardless of flanking residues. Masked variants, with or without MMP2 treatment, were incubated with target cells or receptor domains. In 19 out of 20 cases, MMP2-mediated cleavage restored binding, with affinities reaching 50–100% of the unmasked control (Figure S9). These findings demonstrate that the designed masks effectively block receptor engagement when covalently attached and are fully displaced upon proteolytic cleavage. This study provides the first evidence that cleavable *de novo* designed peptide affinity masks enable robust inhibition and conditional activation of protein binders with therapeutic potential.

### Evaluation of Specificity and Conditional Antagonist Activity of the Lead Candidate EGFRn_mb_M3

To further investigate the masking strategy, we focused on EGFRn_mb due to its potent EGFR antagonist activity and the strong masking performance observed across its designs (Figures 3a and S5a). Among the five variants, we selected mask design M3, which consistently demonstrated an affinity shift of approximately 3 orders of magnitude between its active and inactive forms.

**Figure 3:**
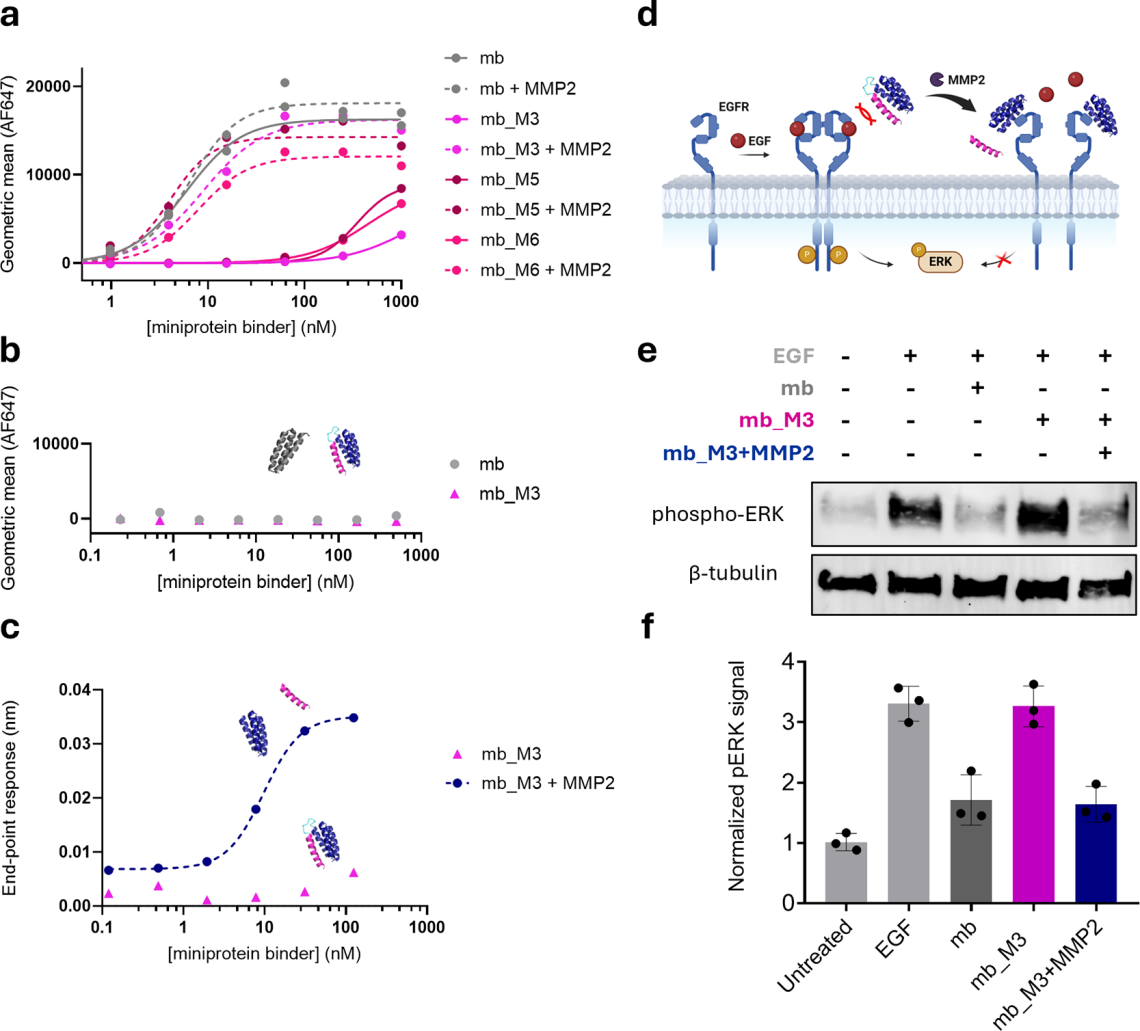
EGFRn_mb masked designs display protease-dependent selective binding and antagonist activity. (a) Flow cytometry analysis of EGFRn_mb_M3 binding, before and after activation, to A-431 cells, which express high levels of EGFR. (b) Flow cytometry analysis of EGFRn_mb_M3 binding, before and after activation, to MCF-7, which express low levels of EGFR. (c) Binding of EGFRn_mb_M3 to EGFR before and after proteolytic activation with MMP2 as measured by biolayer interferometry. (d) Schematic representation of the EGFR antagonist assay with masked mbs. (e) Western Blot analysis of EGFR antagonism assay shows: basal levels of ERK phosphorylation (lane 1), high phospho-ERK levels upon stimulation with EGF (lane 2), inhibition of EGFR signaling with EGFRn (mb) (lane 3), no EGFR signaling inhibition with mb_M3 (lane 4), and full rescue of inhibitory capacity upon MMP2 cleavage (lane 5). (f) Densitometric quantitative analysis of Western Blot (error bars represent the standard deviation, *n* = 3. See Figure S12a,b).

Prior to evaluating the conditional antagonist activity of EGFRn_mb_M3, we validated its targeting and cleavage specificity. Binding assays confirmed that activation restored selective binding to cells with high EGFR expression, while negligible binding was observed in cells with low EGFR levels (Figure 3a,b). A slight increase in masked mb binding at 37 °C compared to 4 °C was noted, potentially due to nonspecific cellular uptake (Figure S10). Additionally, we confirmed that supernatants from cell lines with high levels of active MMP2/9, but not those with low metalloproteinase activity, were able to cleave the mask and generate functional binders (Figure S11) Binding recovery upon cleavage was further confirmed using biolayer interferometry with the EGFR extracellular domain, showing restoration of nanomolar affinity (Figure 3c).

EGFRn_mb specifically targets domain I of EGFR and inhibits its downstream signaling cascade.^48^ To evaluate the functional inhibition exerted by both masked and unmasked forms of the miniprotein binder, we established an assay in which the MAPK pathway was activated via stimulation with epidermal growth factor (EGF), in the presence or absence of the binder (Figure 3d).^27^ We selected the U-87 glioma-derived cell line as a representative tumor model, given its high expression of nonmutated EGFR and its prior use in similar assays.^49,50^ Upon EGF stimulation, we observed an increase in phosphorylated ERK (phospho-ERK) levels, indicating activation of the MAPK pathway (Figures 3e and S12). The unmasked EGFRn_mb (mb) effectively inhibited EGF-induced signaling at two tested concentrations, confirming its antagonist activity. As anticipated, incubation with the masked variant mb_M3 did not result in a reduction of phospho-ERK levels, indicating successful inactivation of the binder. However, when mb_M3 was preincubated with MMP2 protease, its antagonist function was fully restored, enabling it to outcompete EGF and suppress downstream signaling to the same extent as the unmasked binder (Figure 3f). These findings provide compelling evidence that the activity of EGFRn_mb can be reversibly modulated through our masking strategy and reactivated in response to tumor-specific proteases.

### Structural and Biophysical Characterization of Mask–Binder Interactions

EGFRn_mb exemplifies the most common class of *de novo* designed miniprotein binders characterized by a three-helix bundle architecture. These bundles consist of amphiphilic alpha helices, where hydrophobic residues are oriented toward the protein core and hydrophilic residues are exposed to the solvent. The receptor-binding interface involves residues from two helices, while the third helix serves as a structural scaffold. This interface typically features a hydrophobic core surrounded by polar side chains that engage in interactions with the target receptor (Figure S13).

As expected, in the AlphaFold2 and AlphaFold3 predicted complex structure of EGFRn_mb with the mask M3 indicate that binding between the mask and the miniprotein is primarily through shape complementarity and solvent exclusion (Figure 4a). The alpha helical conformation of the mask penetrates the cleft between the two interacting helices and apolar residues on the mask (Leu74, Trp77, Leu85, Tyr84, Leu88 and Leu92) interact with the hydrophobic core of the binder, which is enriched in leucine and aromatic residues, forming a compact four-helix bundle (Figure 4a,b). Additionally, Tyr84 on the mask and Trp52 in the binder engage in a π–π stacking interaction. The hydrophobic inner side of the helical mask contrasts with the polar solvent-exposed side, which is composed of arginine and glutamate residues (Figure 4b). This charged outer shell enables electrostatic interaction between Arg89 in the mask and Glu4 in the miniprotein core (Figure 4a,b). Of the 21 EGFRn_mb residues that interact with the receptor, the mask makes direct contact with 13 and lies within 5 Å of two additional residues. Similar interaction patterns are observed across other mask designs (Figure S13).

**Figure 4:**
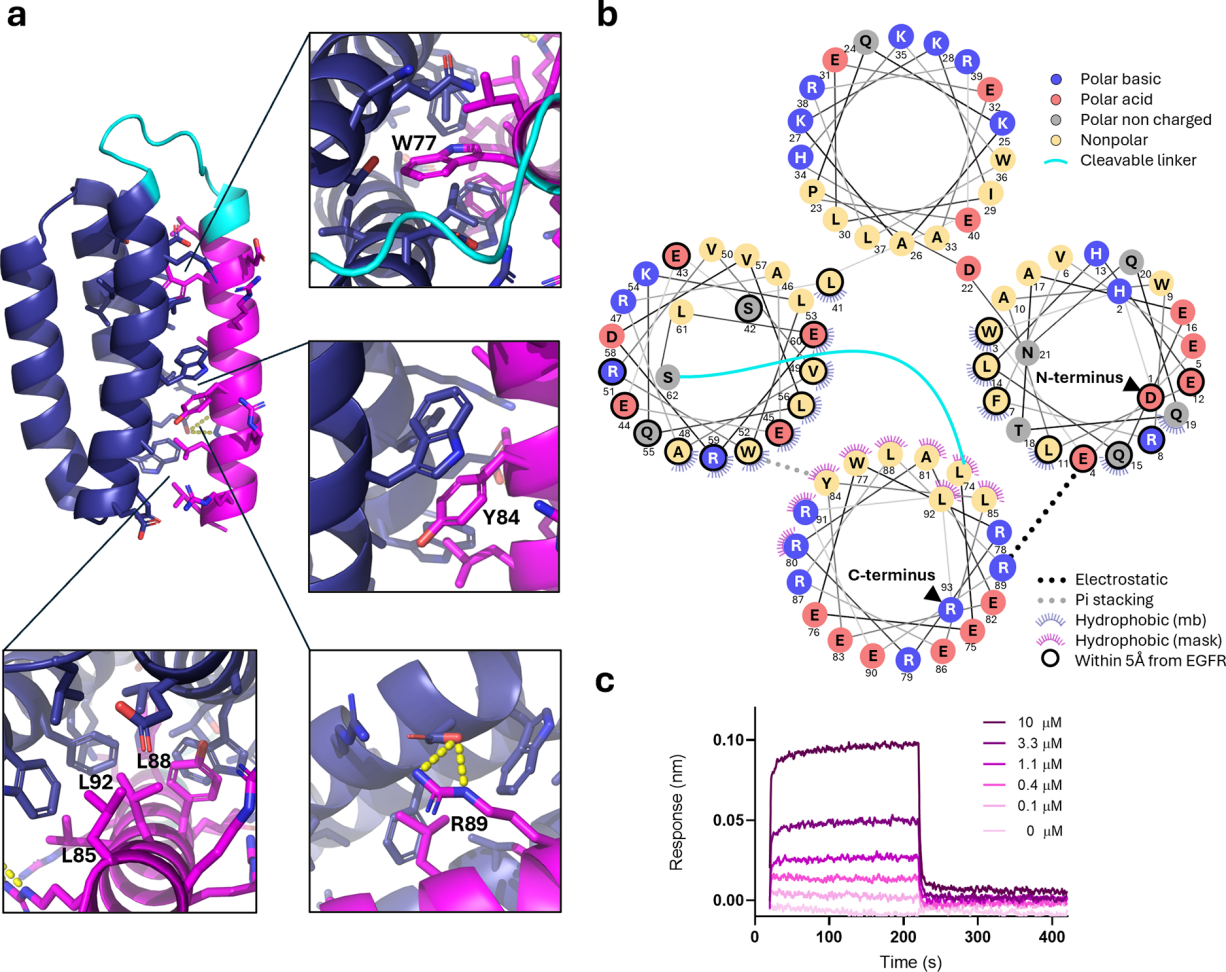
Shape complementarity and solvent exclusion underpin micromolar affinity between M3 and EGFRn_mb, enabling efficient masking and activation. (a) Predicted structure of mb_M3 with AlphaFold 3. Mb core is shown in blue, linker in cyan, and mask in magenta. Residues on the mb or the mask that are within 5 Å from each other are represented as sticks in magenta or in blue, respectively. Polar interactions between the mb and the mask are shown in yellow. In panels, close view of mask residues interacting with miniprotein core. (b) Helical wheel representation of EGFRn_mb and the M3 mask illustrates the coverage of most residues involved in the interaction with EGFR. (c) Biolayer interferometry sensorgrams showing binding of the M3 mask to EGFRn_mb. EGFRn_mb (mb) was immobilized on biosensors and exposed to increasing concentrations of M3. The calculated *K*_D_ is 5 ± 2 μM.

To quantify the affinity of the M3 peptide mask for the miniprotein binder, we produced the mask using Fmoc/*t*Bu solid-phase peptide synthesis (Figure S14). We immobilized the miniprotein binder on a BLI biosensor and studied the binding of the mask. The dissociation constant (*K*_D_) for M3 was determined to be 5 ± 2 μM (Figure 4c). For comparison, we also synthesized the other four mask designs for EGFRn_mb (Figure S14) and all variants displayed micromolar affinities or weaker (Figure S15), confirming that low-affinity interactions are sufficient for effective masking. Although M3 showed the strongest binding and most effective masking, no direct correlation was observed between affinity and masking efficiency across the designs. This likely reflects the fact that peptide masks may also interact with regions outside the receptor-binding interface, contributing to affinity without necessarily enhancing steric blocking.

Kinetic analysis by BLI revealed a *k*_on_ rate for M3 of 5.2 ± 0.1 × 10^4^ M^–1^·s^–1^, comparable to values reported for masks generated via library display technologies. Importantly, this association rate is significantly enhanced when the mask is tethered to the binder due to proximity effects. The measured *k*_off_ rate is 0.33 ± 0.01 s^–1^, which is 10–100 times higher than that of some previously reported masks.^10^ This rapid dissociation facilitates efficient mask removal following protease cleavage, eliminating the need for buffer washes as required for some affinity masks previously described.^5,11,15,17^

### Light Activation of the Masked Miniprotein Binder

Building on the strong performance of EGFRn_mb_M3, we investigated whether its masking strategy could be adapted to respond to alternative stimuli. Specifically, we explored light as a trigger for activation, given its advantages in offering precise spatial and temporal external control compared to proteases, which depend on tissue-specific expression. Unlike protease-cleavable linkers, which can be genetically encoded, light-cleavable linkers require synthetic building blocks not compatible with cellular translation. To overcome this, we devised a chemogenetic strategy to install the synthetic mask, generating a “photocaged” miniprotein binder, mb_PhM3 (Figure 5a). This approach involves encoding a cysteine residue at the C-terminus of EGFRn_mb to enable site-specific conjugation of the mask with a photosensitive peptide linker.

**Figure 5:**
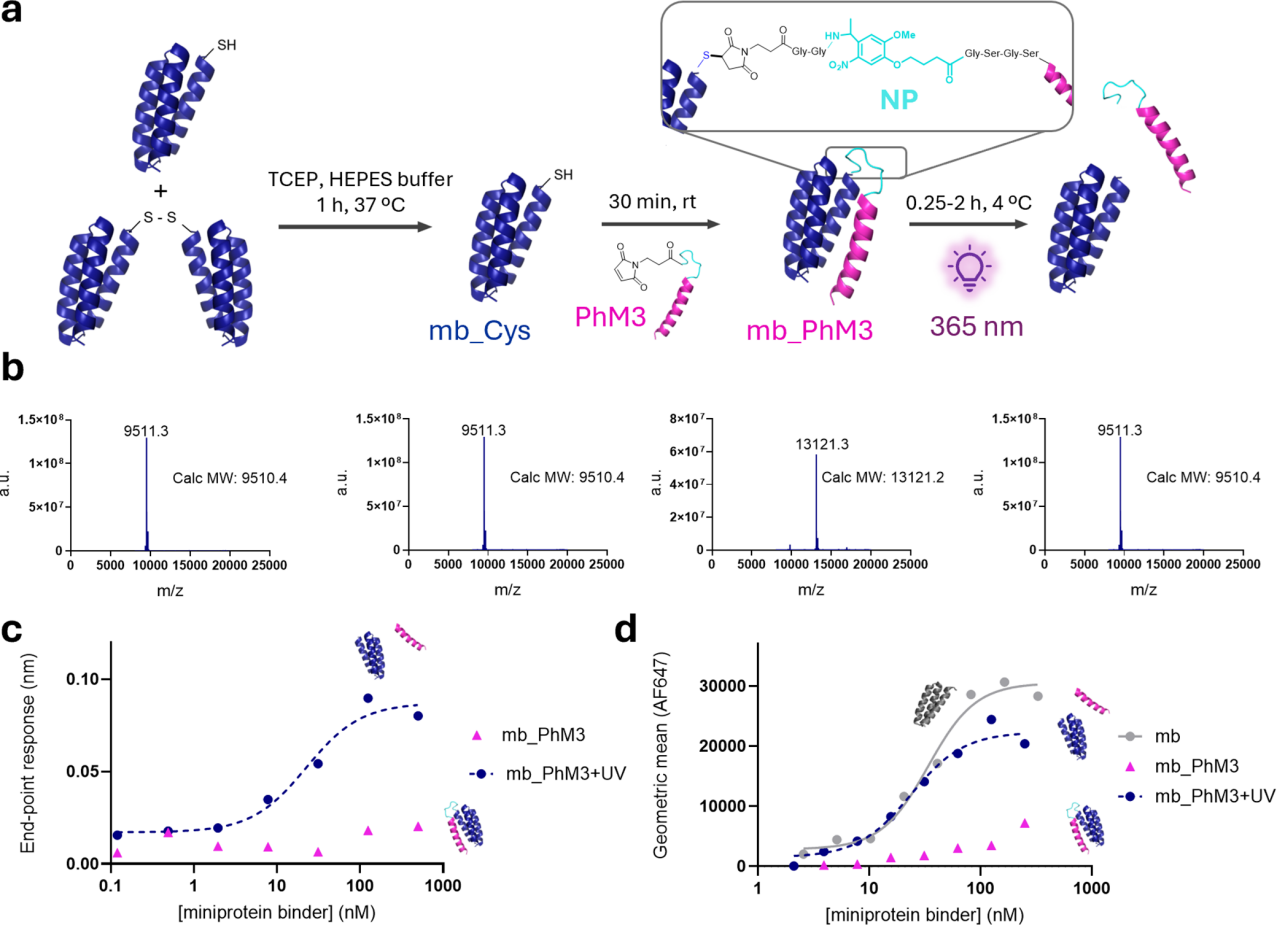
Photocaged EGFRn_mb exhibits robust binding inhibition and full recovery upon light activation. (a) Workflow scheme for production of the photoactivatable miniprotein. A partially dimerized miniprotein with a cysteine residue is reduced with TCEP to give mb_Cys in its monomeric form. The thiol group of mb_Cys is reacted with a maleimide-bearing photocleavable M3 mask (PhM3) to form mb_PhM3, which is cleaved upon UV light irradiation (365 nm). 3-(Maleimido-1-yl)­propanoic and 4-(4-(1-aminoethyl)-2-methoxy-5-nitrophenoxy)­butanoic acid are abbreviated as Mal and NP, respectively. (b) Mass spectra showing, from left to right: mb_Cys as a mixture of dimeric and monomeric forms, reduced monomeric mb_Cys, photoactivated miniprotein (mb_PhM3), and mb_PhM3 after UV light activation for 2 h with 90% of unmasked mb. (c) Binding of mb_PhM3 to EGFR before and after photoactivation as measured by biolayer interferometry. (d) Flow cytometry analysis of mb_PhM3, before and after 0,25–2 h light activation, binding to EGFR on A-431 cells. a.u. arbitrary units.

The photosensitive linker bears a 4-(4-(1-aminoethyl)-2-methoxy-5-nitrophenoxy)­butanoicacid moiety (NP), which undergoes efficient cleavage upon UV irradiation at 365 nm, a wavelength widely used in preclinical applications due to its relatively low toxicity.^51^ Glycine and serine residues flanked the NP group to match the length of the protease-cleavable linker and provide sufficient flexibility for the mask to bind effectively with the miniprotein interacting surface (Figure S16). The photocleavable M3 mask was synthesized via Fmoc/*t*Bu solid phase peptide synthesis (Figure S17) and conjugated to the cysteine-bearing miniprotein. To enhance protein expression, a C-terminal glycine was added (Figure S18). LC–MS analysis revealed partial dimerization of the cysteine-modified miniprotein due to disulfide bond formation (Figures 5a,b and S19). Treatment with TCEP yielded the monomeric form with a free thiol (Figure S20), which was then reacted with an excess of photocleavable M3 mask. Quantitative conjugation was confirmed (Figures 5a,b and S21), and the maleimide ring was found to undergo complete hydrolysis, a modification known to improve linker stability (Figure S22).^50^ The final product, mb_PhM3, was purified using nickel-NTA magnetic beads to maximize protein recovery and verified by RP-HPLC UV chromatogram (Figures 5a,b and S23).

With the photocaged miniprotein binder in hand, we assessed whether binding could be rescued upon UV light irradiation (365 nm). LC-UV-MS confirmed that >85% of the mask is cleaved within 15 min, and >90% after 45 min (Figures 5a,b, S24 and S25). The mass spectrum displayed a dominant peak at 9511 Da, corresponding to the cleaved binder, while the masked construct (13121 Da) was nearly absent. We next demonstrated that the miniprotein binder with the photocleavable mask has negligible binding to the EGFR, as measured by BLI (Figure 5c). The observed reduction in affinity exceeded 2 orders of magnitude, closely matching the performance of the protease-responsive variant. To validate this result in a cellular context, we repeated the experiment by incubating the miniprotein binder with EGFR-overexpressing cells at 4 °C, both before and after light irradiation. Flow cytometry analysis confirmed that light activation fully restored the binding capacity of the parental miniprotein binder, with IC_50_ values comparable to the unmasked control (Figure 5d). These findings indicate that both photocleavable and protease-cleavable masks confer highly effective and reversible inactivation. Importantly, the comparable masking efficiency across linkers with distinct physicochemical properties suggests that the nature of the linker has minimal impact on the functional performance of the masking sequence.

## Conclusion

This study presents a rapid and generalizable strategy for the reversible inactivation of miniprotein binders using minimal peptide masks designed *de novo*. By integrating state-of-the-art computational tools, we engineered cleavable C-terminal extensions that block receptor engagement and can be selectively activated at disease sites. Applied to four therapeutically relevant targets, our approach achieved robust affinity reductions, with nearly half of the 20 designs exceeding 100-fold and the most effective mask reducing EGFR binding by over 3 orders of magnitude. Protease-mediated cleavage restored binding in 19 of 20 cases, confirming the efficiency and reversibility of the masking strategy. The high success rate observed with a small number of tested designs strongly supports the generalizability of this approach and suggests it could be readily extended to many other systems.

Importantly, we demonstrated that micromolar or weaker affinity between the binder and the isolated mask is sufficient for robust inactivation and rapid activation, highlighting the utility of low-affinity interactions that are readily accessible through computational design. The strategy proved compatible with alternative stimuli, as shown by the development of a light-responsive variant using a photocleavable linker, which achieved comparable performance to protease-sensitive designs.

Taken together, our work establishes a streamlined and efficient platform for the *de novo* design of cleavable peptide masks for miniprotein binders. This approach offers a compelling alternative to traditional display-based screening methods and enables the rapid development of conditionally active protein therapeutics responsive to both endogenous and exogenous cues. Building on its versatility and the growing capabilities of computational design, we are actively exploring its broader applicability to other protein formats where conditional activation could enhance therapeutic precision and expand functional control.

## Supplementary Material



## Notes

Detailed experimental procedures, characterization data, supplementary figures and tables (PDF)
