# Supplementary material for: De Novo Design of Peptide Masks Enables Rapid Generation of Conditionally-Active Miniprotein Binders
Source: J Am Chem Soc. 2025 Nov 24;147(49):45495–505. doi: 10.1021/jacs.5c16108 (PMC12703739; doi:10.1021/jacs.5c16108)
Supplement: Supplementary file 1 [file ja5c16108_si_001.pdf]

Supporting Information

De Novo Design of Peptide Masks Enables Rapid Generation of Conditionally-Active Miniprotein Binders

Montserrat Escobar-Rosales,[1‡] Cristina Montaner,[1‡] Marc Expòsit,[2,3] Roberta Lucchi,[1] Cristina Díaz-Perlas,[1] David Baker,[2,3,4] and Benjamí Oller-Salvia[1,*]

[1]Institut Químic de Sarrià (IQS), Universitat Ramon Llull , Via Augusta 390, 08017, Barcelona, Spain

[2]Department of Biochemistry, University of Washington, Seattle, WA, USA

[3]Institute for Protein Design, University of Washington, Seattle, WA, USA

[4] Howard Hughes Medical Institute, University of Washington, Seattle, WA, USA

‡These authors contributed equally to this work.

Table of contents

Experimental section 3

Computational design of miniprotein binders 3

Cloning, expression and purification of miniprotein binders 3

Synthesis and purification of masking peptide sequences 4

Peptide and protein characterization 4

Gel electrophoresis 4

Zymography 4

Liquid chromatography and mass spectrometry analysis (LC-MS) 5

Analytical Size Exclusion Chromatography (SEC) 5

Conjugation of light-sensitive mask 5

Activation of masked miniprotein binders 6

Protease-mediated activation 6

Light-mediated activation 6

Biolayer interferometry (BLI) 6

EGFR vs miniprotein binders 6

EGFRn_mb vs masking peptides 6

Cell assays 7

Cell culture 7

Cell binding assays 7

EGFR antagonist assay 7

MMP2-cleavage in conditioned medium 8

Software used in the manuscript preparation 8

Supplementary Figures 9

Mask Design 9

Masked miniprotein binder expression characterization 10

Activation of masked miniprotein binders 16

Binding assessment of masked and activated miniprotein binders 19

Further characterization of the lead candidate EGFRn mb_M3 21

Activation with different stimulus 27

Supplementary Tables 32

References 38

Experimental section

Computational design of miniprotein binders

To mask the miniprotein binders reported, either RF_{joint} inpainting[37] or RFdiffusion,[21] was used; software is available at https://github.com/RosettaCommons/RFDesign and https://github.com/RosettaCommons/RFdiffusion, respectively. For RFdiffusion (EGFRc, FGFR2, IL7Rα), the hotspot residues to be covered, here the binding interface, need to be specified. The designs were generated and were then fed to ProteinMPNN for sequence redesign of the C-terminal extension. For RF_{joint} inpainting (EGFRn), the model was instructed to template the structure and sequence of the miniprotein binder and build a C-terminal extension comprising: i) the sequence GPLGIAGQ reported to be cleaved by MMP2/9, followed by ii) 15-25 more residues. The model was instructed to inpaint the structure of the protease-sensitive linker and both the structure and the sequence of the terminal 15-25 residues. After generation of the scaffold, the sequences were generated with ProteinMPNN. Finally, the 3D structure of the new designs was predicted with AlphaFold2 (AF2) and its calculated metrics were collected for designs analysis and selection.

The designs generated were filtered based on a series of factors including: i) the predicted Local Distance Difference Test or pLDDT scores predicted by AF2 (complex pLDDT > 90), ii) the Predicted Align Error or PAE of the interaction between the masking helix and the miniprotein binder after removing the linker and predicting the structure of the complex with AF2 (PAE<8), and iii) the number of residues in the interface with the receptor that are within 5 Å of the mask in the predicted structures of the designs. Residue coverage thresholds used to narrow down the list to 20-40 designs were: 75% for EGFRn, 65% for EGFRc, 60% for FGFR2, and 80% for IL7Rα. Five designs for each mb were selected based on sequence and length diversity.

Cloning, expression and purification of miniprotein binders

The DNA sequences encoding for the 5 selected designs and the unmasked EGFRn_mb control with an N-terminal His-tag were cloned via restriction enzyme digestion cloning into pET29b (Table S1-S2), using *NdeI* and *XhoI* restriction enzymes (New England Biolabs, Ipswich, MA, USA), while the DNA sequences of EGFRc, FGFR2 and IL7Rα miniprotein binders encoding for the 5 selected designs and the unmasked control with an N-terminal His-tag were cloned via Golden Gate Assembly into pET29b (Table S1-S2), using *BsaI* restriction enzyme (New England Biolabs, Ipswich, MA, USA). Constructs' expression was carried out in LB + 50 mg/mL kanamycin, inducing with 0.5 or 1 mM IPTG when OD_{600} had reached at least 0.8, and expressing overnight at 30°C. On the following day, cells were harvested centrifuging for 15 min at 4°C and >4000 g. Pellets were resuspended in lysis buffer (50 mM Tris-HCl pH 8, 150mM NaCl, 1% Triton X-100 and 1 mM PMSF) and sonicated at 40% amplitude, with 20 s ON and 30 s OFF pulses, for a total of 10 min on ice. Lysate was clarified centrifuging at 20000 g for 20 min at 4°C. After filtering the supernatant through a 0.22 μm filter, the miniprotein binders were purified via Immobilized Metal Chelate Affinity Chromatography (1 mL IMAC HisTrap™ HP column, Cytiva, Amersham, UK) on a fast protein liquid chromatography system (FPLC, BIO-RAD NGC, BIO-RAD, Hercules, CA, USA). Purification was carried out in 50 mM Tris-HCl pH=8.0 and 50 mM NaCl, ensuring protein elution with 500 mM imidazole. After purification, imidazole was removed by dialysis against 50

mM Tris-HCl pH 8 and 50 mM NaCl and proteins were concentrated using 4 mL Amicon Ultra concentrators with 3 kDa MWCO.

Synthesis and purification of masking peptide sequences

All peptides were synthesized with an automated peptide synthesizer using microwave assisted Liberty Blue™ 2.0 (CEM Corporation) using 9-fluorenylmethyloxycarbonyl (Fmoc) chemistry and L-amino acids and a Fmoc-Rink-Amide resin (Iris Biotech, BR-1340, substitution of 0.65 mmol/g). All amino acids and coupling reagents were dissolved in DMF. Amino acids are sequentially conjugated with cycles consisting of 1 min deprotection (10% Piperidine at 90°C) and a 2 min coupling reaction (4 eq of amino acid, 4 eq of DIC (N,N'-Diisopropylcarbodiimide) and 4 eq of OxymaPure®). For Arg and Photolinker (PhL)-containing amino acids, coupling was performed twice. The conjugation of Mal-COOH (Iris Biotech, PEG2135) was performed manually in solid phase, activating with DIC (4 eq) and OxymaPure® (4 eq). To confirm that Mal-COOH had been successfully incorporated, a ninhydrin test was performed.

Peptides were cleaved with concomitant removal of the side chain-protecting groups in a cleavage cocktail made of trifluoroacetic acid (TFA), water and triisopropylsilane (TIS) in the following proportions 95:2.5:2.5 (v/v). For peptides containing the photolinker moiety, a 50% dichloromethane was added to lower the percentage of final TFA to 45%. Peptides were precipitated with diethyl ether, solubilized in a mixture of H_2O/ACN (50:50), and lyophilized. Peptides were resuspended in a mixture of water and up to 30% ACN. Purification was carried out with preparative RP-HPLC using an Aeris Peptide XB-C18 100 LC Column (250 x 21.2 mm, 5 µm, Phenomenex) mounted on an Agilent 1260 Infinity II system with ChromoScope software (1260 DAD WR, 1260 Vial Sampler and 1260 Flexible Pump). Purification was conducted in a 20-70% gradient of acetonitrile (0.1% TFA) in water (0.1% TFA), with a flow of 20 mL/min. The fractions containing the peptide were identified using the LC-MS, pooled together and lyophilized.

Peptide and protein characterization

Gel electrophoresis

SDS-PAGE was performed using a BioRad Mini-PROTEAN® Tetra cell system with in-house produced gels containing 15% acrylamide or commercial Any kD™ Mini-PROTEAN® TGX Stain-Free™ Protein Gels. SDS-PAGE gels were run at 180 V for 40 min in electrophoresis buffer (25 mM Tris-base, 190 mM glycine, 3.5 mM SDS). Gels were stained with a Coomassie solution (10% glacial acetic acid and 0.25 g/L brilliant blue) and destained with a destaining solution (20% methanol and 3% glacial acetic acid in water). Gels were visualized using the ChemiDoc™ MP Imaging System (Bio-Rad, 12003154) with the 590/110 nm standard filter using Trans-white illumination.

Zymography

Gelatin zymography was performed to assess extracellular proMMP-2 and MMP-2 activity levels. Briefly, 10 µL of culture medium was subjected to SDS–polyacrylamide gel electrophoresis (SDS-PAGE) using a 10% polyacrylamide gel containing 0.1% gelatin (Thermo Fisher, ZY00105BOX). Following electrophoresis, gels were incubated at room temperature for 30 minutes in Zymogram Renaturing Buffer (Thermo Fisher Scientific, Cat# LC2670) to allow protein renaturation. Subsequently, gels were incubated at 37°C for 20 hours in Zymogram Developing Buffer (Thermo Fisher Scientific, Cat# LC2671) to enable enzymatic digestion of the gelatin substrate. Gels were then stained with 0.1% Coomassie Brilliant Blue R-250 and destained in a solution containing 10% acetic acid and 30% methanol

in water. Gelatinolytic activity was visualized as clear bands against a blue background, corresponding to areas where gelatin was degraded by active MMPs.

Liquid chromatography and mass spectrometry analysis (LC-MS)

Samples were analyzed by mass spectrometry with analytical HPLC Agilent 1260 Infinity II system coupled to the mass-spectrometer Agilent 6230 LC/TOF (Model G6230B). For peptide characterization, samples (ca. 200 ng of peptide) were injected into an Aeris 5 µm PEPTIDE XB-C18 150 x 4.6 mm (Phenomenex, 00F-4632-E0), applying a 20-75% gradient of acetonitrile (0.1% formic acid) in water (0.1% formic acid) over 10 min at a flow rate of 0.8 mL/min at 30°C. Proteins (ca. 400 ng) were injected into a bioZen 2.6 µm Wide Pore C4 100 x 2.1 mm LC column (Phenomenex, 00D-4786-AN), applying a 20-70% gradient of acetonitrile (0.1% formic acid) in water (0.1% formic acid) over 10 min at a flow rate of 0.3 mL/min at 80°C. Peptides were analyzed at 220 nm while proteins at 280 nm. The TOF-MS was operated in positive ion mode over a m/z range of 300–3200 Da, with a cone voltage of 4000 V and a sheath gas temperature of 325°C. Data acquisition was done with the Agilent MassHunter Workstation Data Acquisiton software, while data evaluation was performed using Bioconfirm 10.0, where spectra were deconvoluted to a mass range of 8-20 kDa.

Analytical Size Exclusion Chromatography (SEC)

Samples of EGFRc, FGFR2 and IL7Rα miniproteins were analyzed with analytical HPLC Agilent 1260 Infinity II system, while EGFRn miniproteins were analyzed using the fast protein liquid chromatography system (FPLC, BIO-RAD NGC, BIO-RAD, Hercules, CA, USA). In both cases, the standard (BEH200 SEC Protein Standard Mix, Waters, 186006518) and samples were injected into a Bioresolve™ SEC mAb column, 200 Å, 2.5 µm, 4.6 x 150 mm (Waters, 186009435), applying 100% 150 mM ammonium acetate pH 6.9, over 10 min, 30°C, at a flow rate of 0.250 mL/min (HPLC) and 0.4 mL/min (FPLC). EGFRn and IL7Rα miniproteins were analyzed at 280 nm while EGFRc and FGFR2 miniproteins were analyzed at 220 nm due to the absence of tryptophan in their sequences. In HPLC, data acquisition was done with the Agilent MassHunter Workstation Data Acquisition software and data evaluation was performed using Bioconfirm 10.0. In FPLC, data acquisition was done using the ChromLab software version 6.1.27.0 (Bio-Rad Laboratories, Inc)

Conjugation of light-sensitive mask

Miniprotein binder bearing an encoded cysteine (mb_Cys) was found to be partly dimerized by a disulfide bond. Reduction of the dimer was achieved by the addition of 10 eq of Tris-(2-carboxyethyl)-phosphine (TCEP) for 1h at 37°C, in 10 mM HEPES, 150 mM NaCl, pH 7.4 buffer. Conjugation of the miniprotein binder to the maleimide-bearing mask was achieved with the addition of 20 eq of the peptide, without need of prior removal of the TCEP. Conjugation was monitored by LC-MS. To remove the excess peptide and TCEP, His-tagged miniprotein binders were captured in Pierce™ High-Capacity Ni-IMAC Magnetic Beads (A50588) and eluted with a 500 mM imidazole solution. Dialysis was performed to eliminate the excess of imidazole.

Activation of masked miniprotein binders

Protease-mediated activation

Cleavage of the mask from the miniprotein binder was carried out *in vitro* using pro-MMP2 (10082-HNAH, Recombinant Human MMP2 Protein, HPLC-verified, Sino Biological, Beijing, China). The enzyme at 1.2 mM was first activated with 2.5 mM of 4-Aminophenylmercuric acetate (APMA, Sigma Aldrich) in TTC buffer (Tris-triton-calcium: 50 mM Tris-HCl pH 7.5, 1 mM CaCl2, 0.05% Triton X-100) for 2h at 37°C. After activation, the enzyme was added to 20 µM of miniprotein binder to reach a final concentration of 160 or 80 nM (250:1 or 125:1 miniprotein binder:enzyme). Proteolysis was stopped adding MMP2 inhibitor (SB-3CT, 1 µM). The cleavage was carried out in TTC buffer at 37°C for up to 2h and the cleavage percentage analyzed by HPLC.

Light-mediated activation

The photo-sensitive miniprotein binder (mb_PhM3) was cleaved by exposure to UV light at a wavelength of 365 nm from a UV lamp at a 10 cm distance at 4°C. The measured irradiance was 1.7 mW/cm^2. Samples were taken at several time points and the cleavage analyzed by LC-MS. To ensure complete cleavage, the UV light irradiation was left for 2h, but at 15 min almost complete cleavage was confirmed by LC-MS.

Biolayer interferometry (BLI)

Binding assays were performed on Octet R2 (Sartorius). Experiments were designed and executed from the Octet BLI Discovery 13.0 software. Titrations were performed at 30°C while rotating at 1000 rpm.

EGFR vs miniprotein binders

To assess miniprotein binders' affinity to EGFR, biotinylated EGFR (2 µg/mL, EGFR-b 10001-H27H-B, Sino Biological) was loaded onto streptavidin-coated biosensors (SA biosensors, 18-5019) in binding buffer (10 mM HEPES pH 7.4, 150 mM NaCl, 0.05% Tween-20, 1 mg/mL BSA) until 1 nm immobilization was reached. In the association phase, the sensors were dipped into miniprotein binder solutions at several concentrations (1-1000 nM) in binding buffer for 200 s. Then, for the dissociation phase, the biosensors were dipped into binding buffer for 200 s. After each cycle, biosensors were regenerated by 4 cycles of dipping the sensor in a HCl pH 0.5 solution for 5s and binding buffer for 5s. End-point data was extracted from BLI sensorgrams and plotted against protein concentration. Data was analyzed using GraphPad software.

EGFRn_mb vs masking peptides

To assess masking peptides' affinity to EGFRn_mb, miniprotein binder was immobilized in amine-reactive biosensors (AR2G biosensors, 18-5092). Biosensors were first activated with 20 mM EDC (1-Ethyl-3-[3-dimethylaminopropyl] carbodiimide hydrochloride) and 10 mM s-NHS (N-hydroxysulfosuccinimide), and then dipped in 20 µg/mL of EGFRn_mb in 10 mM acetate buffer pH 5) for 800 s, followed by the quenching of the remaining activated positions in 1M ethanolamine. A baseline was established in binding buffer alone (PBS pH 7.4, 0.05% Tween-20, 1 mg/mL BSA). In the association phase, the sensors were dipped into the peptide solutions at several concentrations (1-10000 nM) in binding buffer for 200 s. Then for the dissociation phase, the biosensors were dipped into binding buffer for 200 s. Kinetic data were collected and processed using a 1:1 binding model to obtain the affinity constants using Octet Analysis Studio 13.0.

Cell assays

Cell culture

Mycoplasma-free U-87, MCF-7, A-431, A-549, SK-BR-3, HeLa and SK-OV-3 cells were cultured in DMEM High Glucose medium (BioWest, Nuaillé, France) supplemented with 10% fetal bovine serum (FBS, VWR Life Science Seradigm), 2 mM L-glutamine (BioWest, Nuaillé, France) and antibiotics (60 and 100 mg/L of penicillin and streptomycin respectively, Biowest, Nuaillé, France). Cells were cultured in T75 flasks until 80% confluence at 37°C and 5% CO2, then passaged. All work performed with human cells follow the ethical principles and EU Directive 2004/23/EC.

Cell binding assays

A-431 and MCF-7 cells were seeded at a density of 20000 cells/well in 96-well plates. Miniprotein binders' dilutions for the assay were prepared in PBS + 1% Bovine Serum Albumin (BSA, Sigma Aldrich, Milwaukee, WI, USA). Cell binding assays were performed 48 h after seeding as follows. After washing the medium, cells were incubated with the miniprotein binders at different dilutions (50 mL/well) for 1h on ice. Then, unbound minibinder were washed away with PBS and cells were incubated with Alexa Fluor® 647 anti-His Tag Antibody (BioLegend, San Diego, CA, USA) at 1:1500 dilution in PBS+1% BSA for 45 min on ice. Upon washing away the secondary antibody solution, cells were detached with 25 mL/well of Trypsin-EDTA (Gibco) for 5 min at 37°C, and trypsin was inactivated by adding 200 μL/well of complete DMEM with 33% (v/v) formalin. Cells were analyzed with an Agilent NovoCyte Flow cytometer, detecting at 640 nm the cells with AF647-conjugated antibody on the surface. Data was analyzed plotting the geometric mean of AF647+-cells against the concentration of the miniprotein binder.

EGFR antagonist assay

U-87 cells expressing EGFR were seeded in 12-well plates until confluency was reached. After that, supplemented DMEM medium was aspirated, and cells were washed twice with PBS. Then, DMEM serum-free medium was added, and U-87 cells were serum-starved for 48 h. Following the incubation period, the starvation medium was aspirated, and cells were treated with either 100 nM or 1000 nM of the corresponding miniprotein binder (mb, mb_M3 or mb_M3+MMP2) for 1 h at 37°C, 5% CO_2. Right after this, cells were stimulated with EGF 1 nM (PHG0315, Gibco) for 15 min at 37°C, 5% CO_2. Afterwards, the medium was aspirated, and cells were washed once with cold PBS before the lysis treatment.

For total protein isolation, U-87 cells were lysed using Pierce™ RIPA buffer (ThermoFisher Scientific, Rockford, IL, USA) supplemented with 7X cOmplete Mini-EDTA free serine/ cysteine protease inhibitor cocktail (Roche Diagnostics GmbH, Mannheim, Germany). All cell-lysate supernatants were collected in microcentrifuge tubes and quantified using the Pierce ™ BCA Protein Assay kit (ThermoScientific). After this, 4X Laemmli sample buffer (Bio-Rad), supplemented with 10% β-mercaptoethanol, was added to all samples before being heated at 95°C for 5 min. Cell lysates were resolved by SDS-PAGE, transferred to low PVDF membranes and blotted using the Trans-blot Turbo Mini Transfer kit (Bio-Rad). The membranes were blocked in TBS-T (Tris Buffer Saline: 50 mM Tris, 138 mM NaCl, 2.7 mM KCl, pH 8.0 + 0.1% Tween-20) with 5% BSA for 1 h and then, incubated overnight at 4°C, under constant stirring, with the Phospho-p44/p42 MAPK Erk 1/2 (1:1000, Cell Signaling Technologies) and β-tubulin (1:1000, Cell Signaling Technologies) primary antibodies. The next day, membranes were washed three times with TBS-T before being incubated with Goat Anti-

Rabbit IgG StarBright Blue 700 (1:2500, Bio-Rad) and Goat Anti-Mouse IgG StarBright Blue 520 (1:2500, Bio-Rad) secondary antibodies, respectively, for 1 h at room temperature. Finally, the membranes were washed five times with TBS-T and visualized using the ChemiDoc MP Imaging System System. Densitometric analysis of membranes was carried out using the Image Lab software 6.1 (Bio-Rad).

MMP2-cleavage in conditioned medium

Conditioned media were collected from confluent T25 flasks of A-549, A-431, HeLa, U-87, and SKOV3 cells. Miniprotein binders were diluted to a final concentration of 1000 nM in the collected media and incubated for 24 hours at 37°C to allow MMP2-mediated proteolytic processing. Cleavage was confirmed by SDS-PAGE.

Software used in the manuscript preparation

Protein structures were visualized using PyMOL v3.1.6.1, and all graphical data representations were generated with GraphPad Prism v10. Figure 3d was created with BioRender.com. Copilot was employed to assist in refining the manuscript's writing.

Supplementary Figures

Mask Design

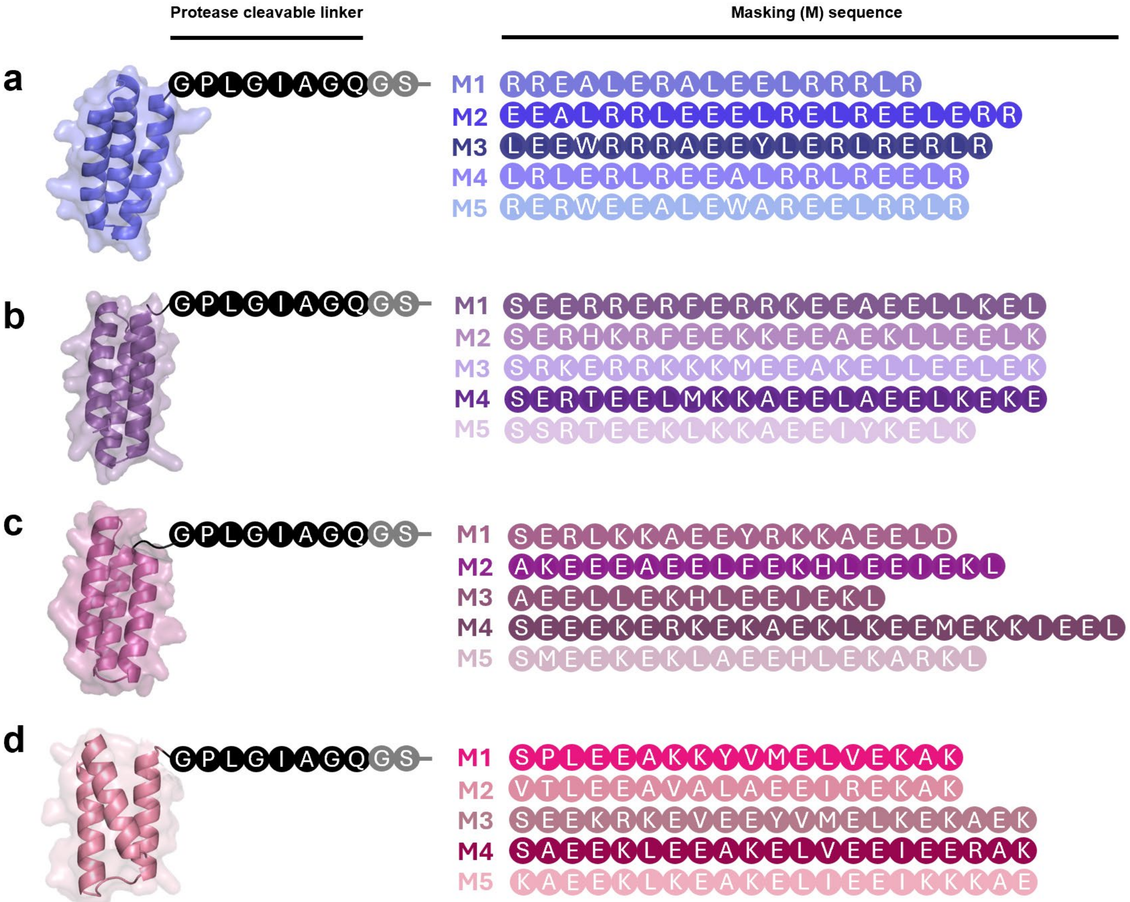

Figure S1. Sequences of the selected masked miniproteins. The masks generated by RFDiffusion are comparable to those described here for RFjoint. a) M1-M5 masks of EGFRn_mb; b) M1-M5 masks of EGFRc_mb; c) M1-M5 masks of FGFR2_mb; c) M1-M5 masks of IL7Rα_mb.

Masked miniprotein binder expression characterization

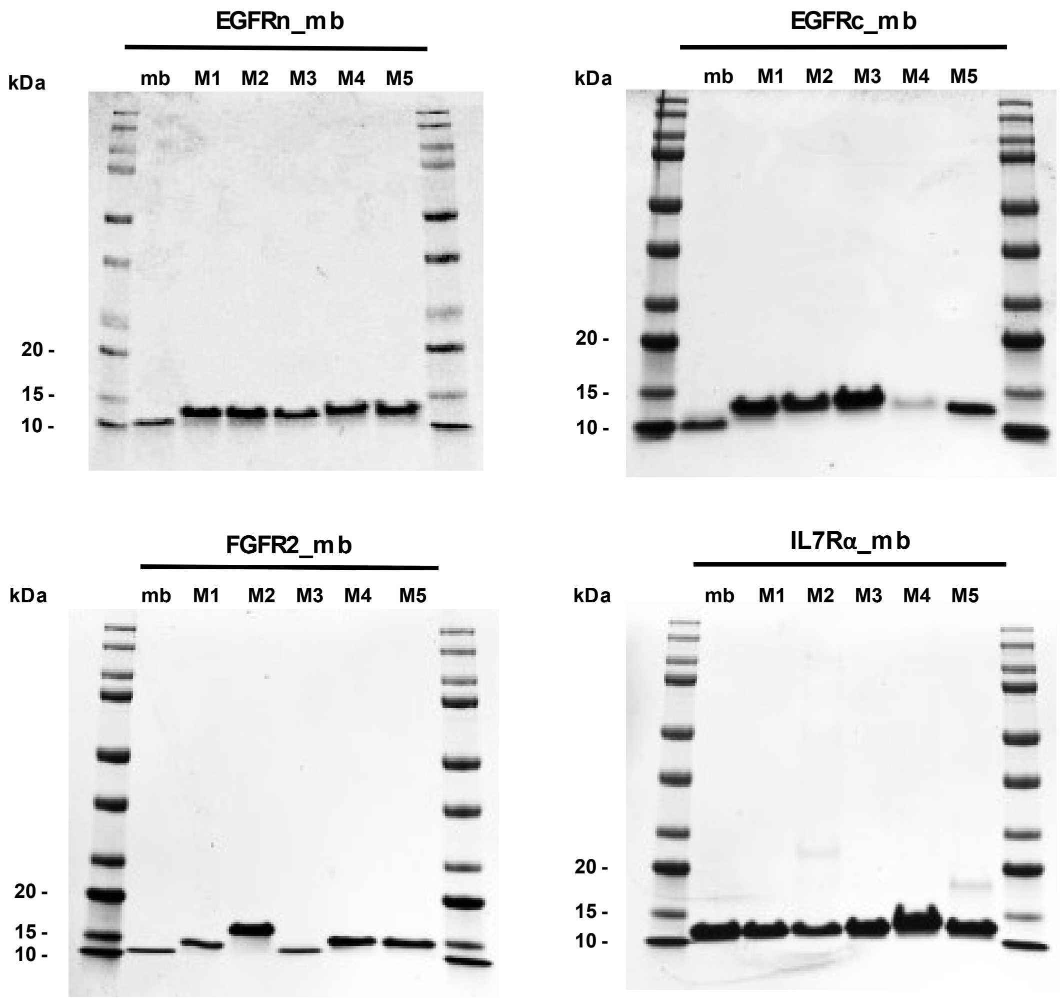

Figure S2. Characterization of expressed miniproteins. Coomassie blue stained SDS-PAGE of expressed masked miniproteins.

a

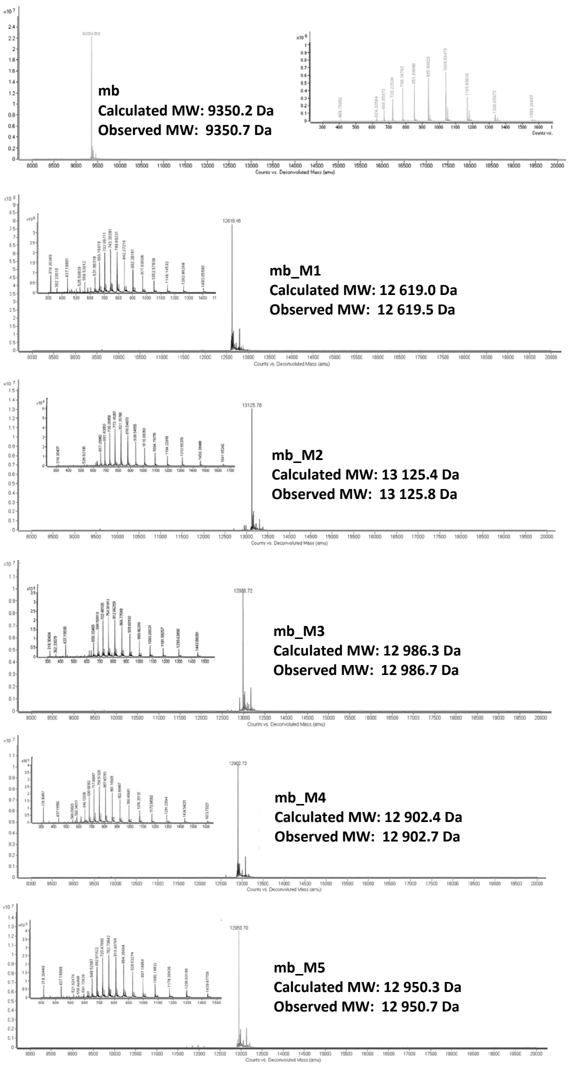

mb
Calculated MW: 9350.2 Da
Observed MW: 9350.7 Da
mb_M1
Calculated MW: 12 619.0 Da
Observed MW: 12 619.5 Da
mb_M2
Calculated MW: 13 125.4 Da
Observed MW: 13 125.8 Da
mb_M3
Calculated MW: 12 986.3 Da
Observed MW: 12 986.7 Da
mb_M4
Calculated MW: 12 902.4 Da
Observed MW: 12 902.7 Da
mb_M5
Calculated MW: 12 950.3 Da
Observed MW: 12 950.7 Da

b

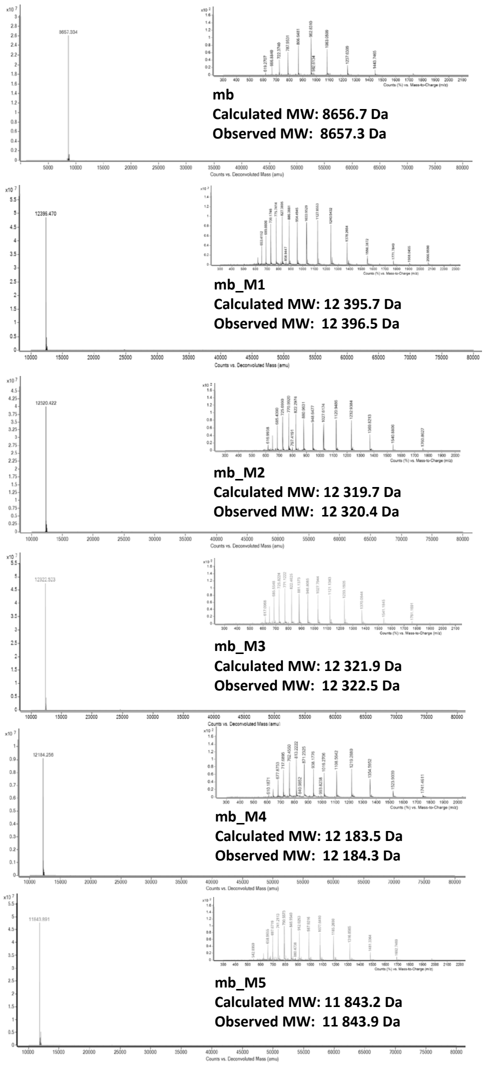

mb
Calculated MW: 8656.7 Da
Observed MW: 8657.3 Da
mb_M1
Calculated MW: 12 395.7 Da
Observed MW: 12 396.5 Da
mb_M2
Calculated MW: 12 319.7 Da
Observed MW: 12 320.4 Da
mb_M3
Calculated MW: 12 321.9 Da
Observed MW: 12 322.5 Da
mb_M4
Calculated MW: 12 183.5 Da
Observed MW: 12 184.3 Da
mb_M5
Calculated MW: 11 843.2 Da
Observed MW: 11 843.9 Da

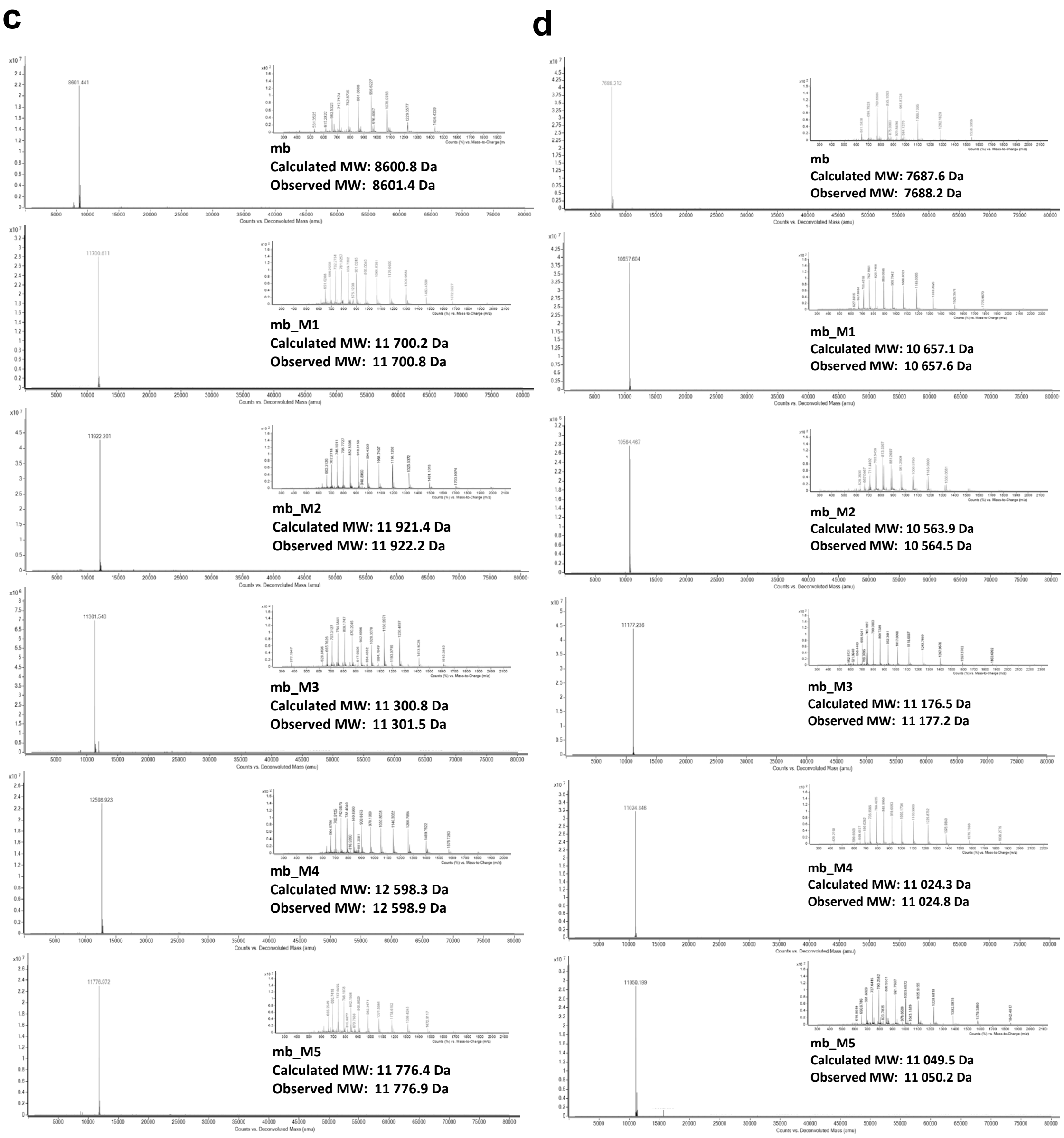

Figure S3. Characterization of expressed miniproteins. All panels present both non-deconvoluted and deconvoluted mass spectrometry spectra. Data is shown for the unmasked (mb) and masked (M1-M5) miniproteins of a) EGFRn_mb, b) EGFRc_mb, c) FGFR2_mb, and d) IL7Rα_mb.

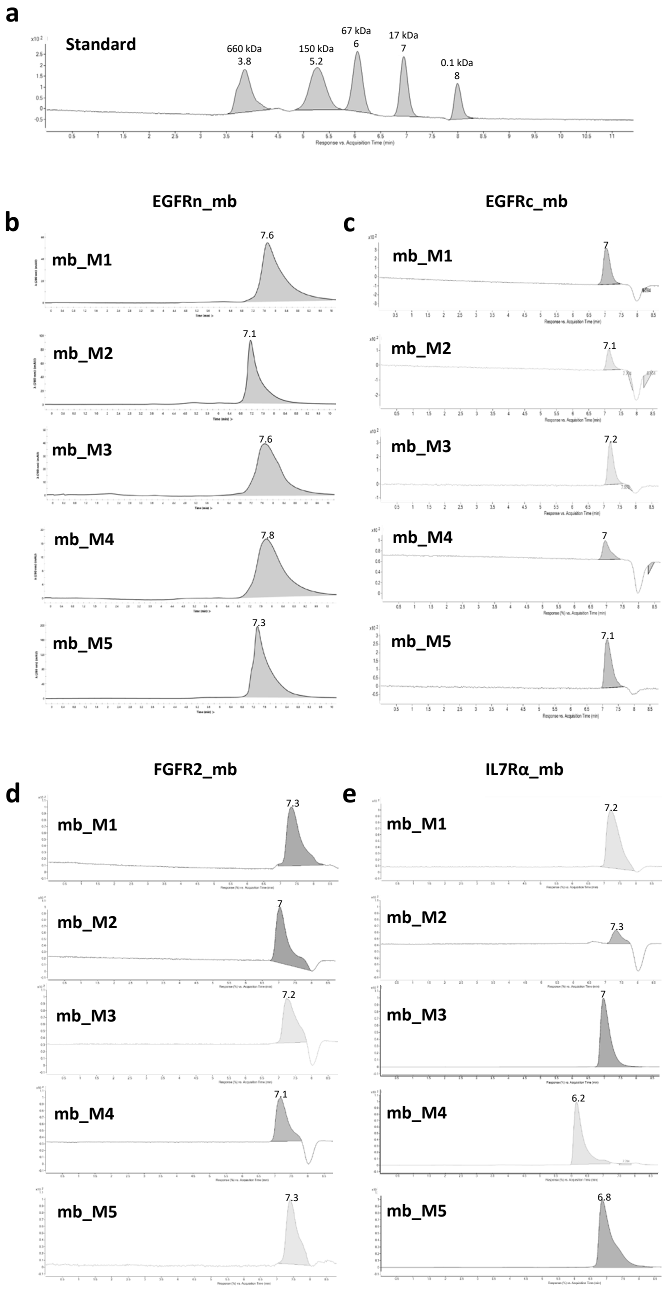

Figure S4. Analytical Size Exclusion Chromatography (SEC) of expressed miniproteins. a) Standard using the analytical HPLC Agilent 1260 Infinity II system; b) EGFRn_mb, c) EGFRc_mb; d) FGFR2_mb and e) IL7Rα_mb constructs. The panels display UV chromatograms at 220 nm for EGFRc_mb and FGFR2_mb (does not contain tryptophan in their sequences) and at 280 nm for EGFRn_mb and IL7Rα_mb.

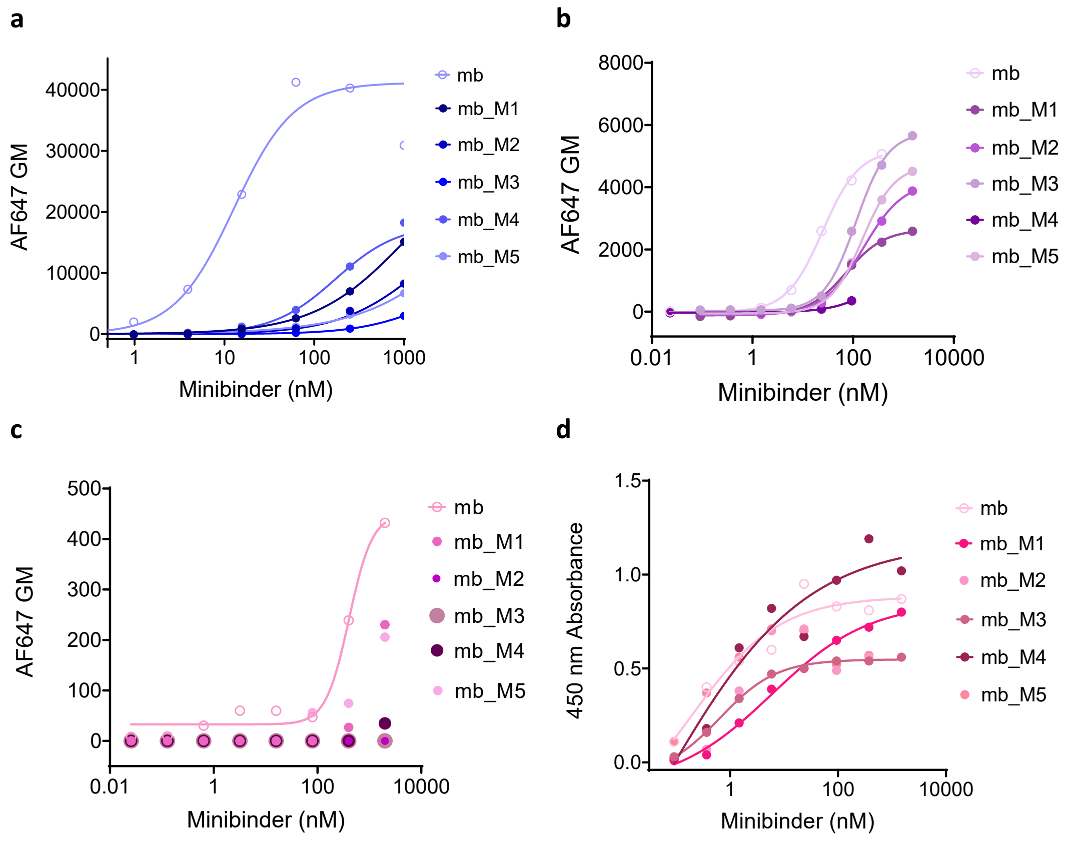

Figure S5. Binding inhibition of masked constructs. The masking level of all selected masked miniproteins compared to the unmasked version (mb) was obtained using Flow Cytometry. Data is shown for a) EGFRn_mb, b) EGFRc_mb, c) FGFR2_mb and d) IL7Rα_mb.

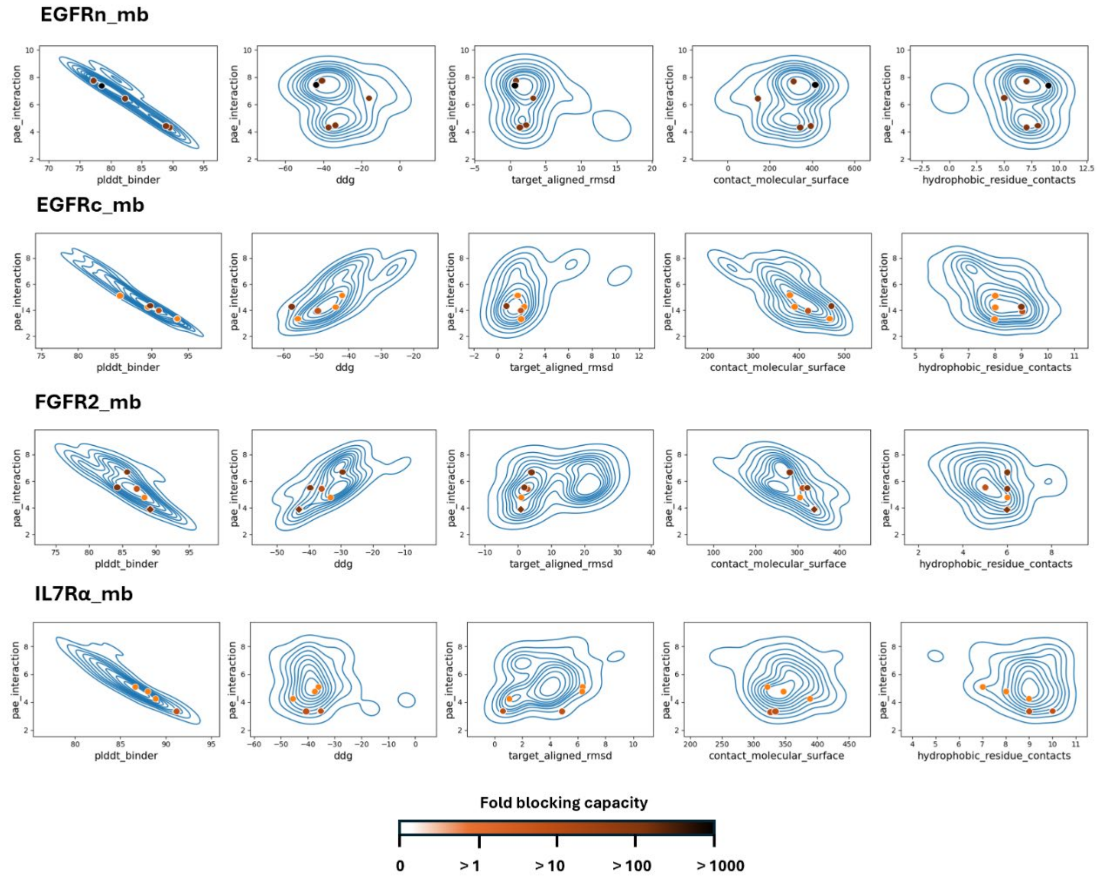

Figure S6. Correlations between Rosetta interface metrics and AlphaFold2 parameters for the selected miniprotein masks. Scatter plots include all designs generated using the AI-driven workflow for EGFRn, EGFRc, FGFR2, and IL7Rα, filtered by pLDDT and PAE as described in the main text. Circles indicate designs selected for experimental validation, with blocking capacity represented by a color gradient. All designs were activatable by MMP-2 except the one shown as a diamond.

Activation of masked miniprotein binders

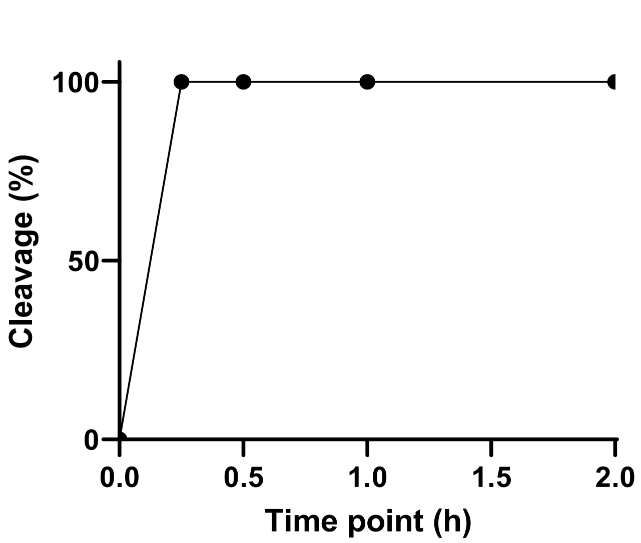

Figure S7. Cleavage kinetics of EGFRn mb_M3 in the presence of MMP-2. MMP-2 cleavage was performed under the conditions described in the methods section. After 5 min of incubation with this metalloprotease, EGFRn_mb M3 was completely cleaved. MMP-2 activity was determined with EnzChek® Gelatinase/Collagenase Assay Kit (Molecular Probes, Inc.). Upon comparison with the Collagenase standard, the found activity of MMP-2 was 1.4 U/mL (One unit is defined as the amount of enzyme required to liberate 1 µmol of L-leucine equivalents from collagen in 5 hours at 37°C, pH 7.5).

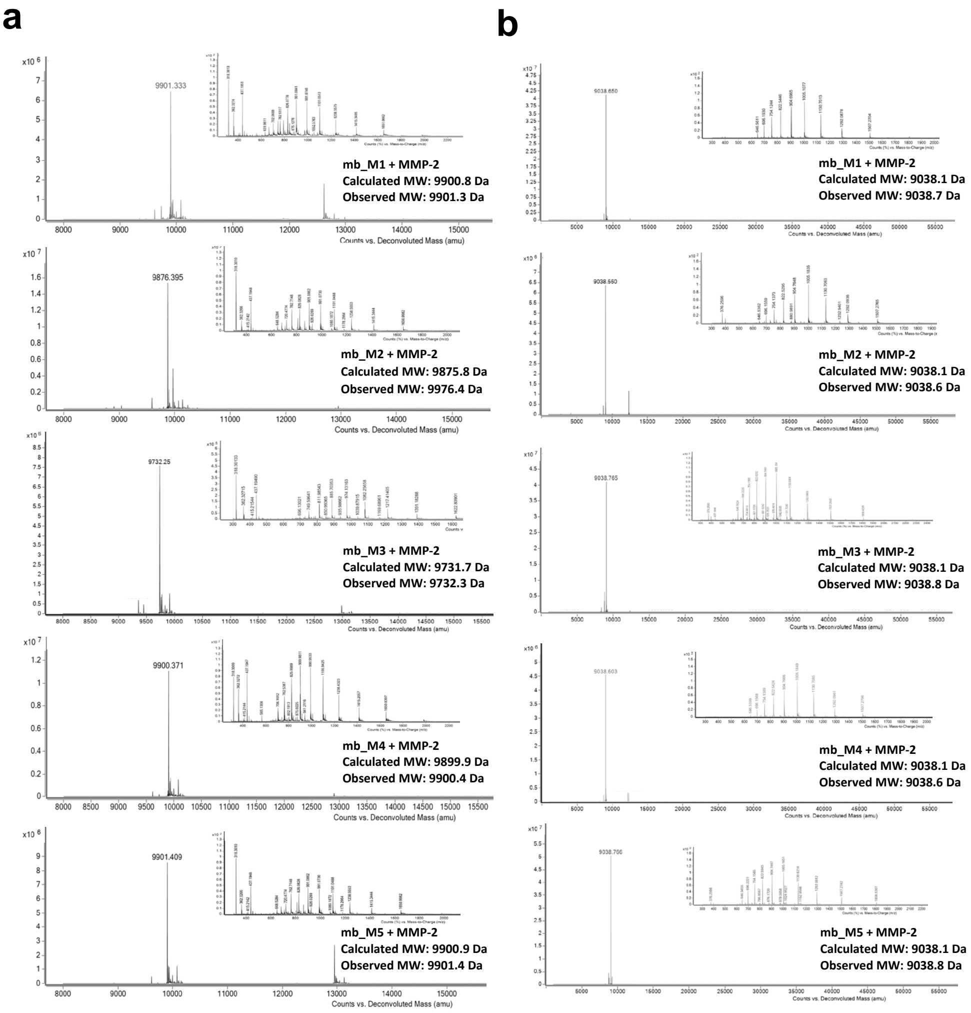
a
b
mb_M1 + MMP-2
Calculated MW: 9900.8 Da
Observed MW: 9901.3 Da
mb_M2 + MMP-2
Calculated MW: 9875.8 Da
Observed MW: 9976.4 Da
mb_M3 + MMP-2
Calculated MW: 9731.7 Da
Observed MW: 9732.3 Da
mb_M4 + MMP-2
Calculated MW: 9899.9 Da
Observed MW: 9900.4 Da
mb_M5 + MMP-2
Calculated MW: 9900.9 Da
Observed MW: 9901.4 Da
mb_M1 + MMP-2
Calculated MW: 9038.1 Da
Observed MW: 9038.7 Da
mb_M2 + MMP-2
Calculated MW: 9038.1 Da
Observed MW: 9038.6 Da
mb_M3 + MMP-2
Calculated MW: 9038.1 Da
Observed MW: 9038.8 Da
mb_M4 + MMP-2
Calculated MW: 9038.1 Da
Observed MW: 9038.6 Da
mb_M5 + MMP-2
Calculated MW: 9038.1 Da
Observed MW: 9038.8 Da

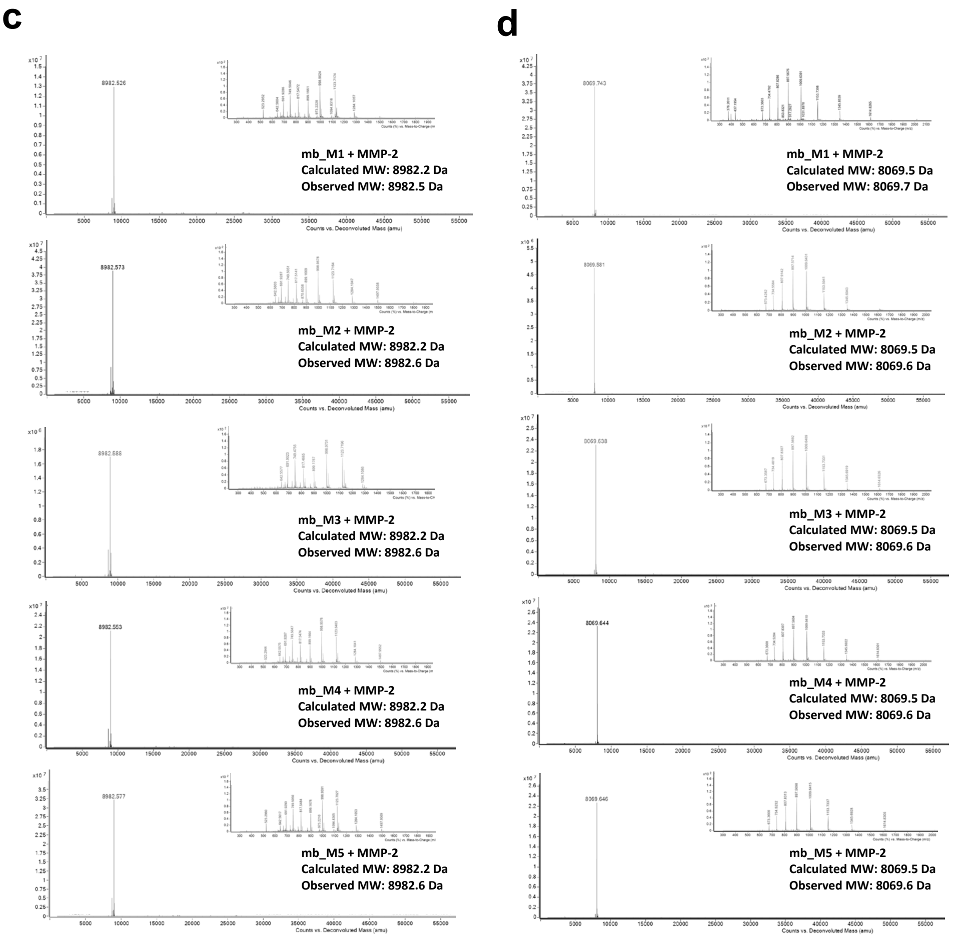

Figure S8. Characterization of masked miniproteins after MMP-2 cleavage. All panels present both non-deconvoluted and deconvoluted mass spectrometry spectra. Data of cleaved a) EGFRn, b) EGFRc, c) FGFR2, and d) IL7Rα miniproteins is shown.

Binding assessment of masked and activated miniprotein binders

a

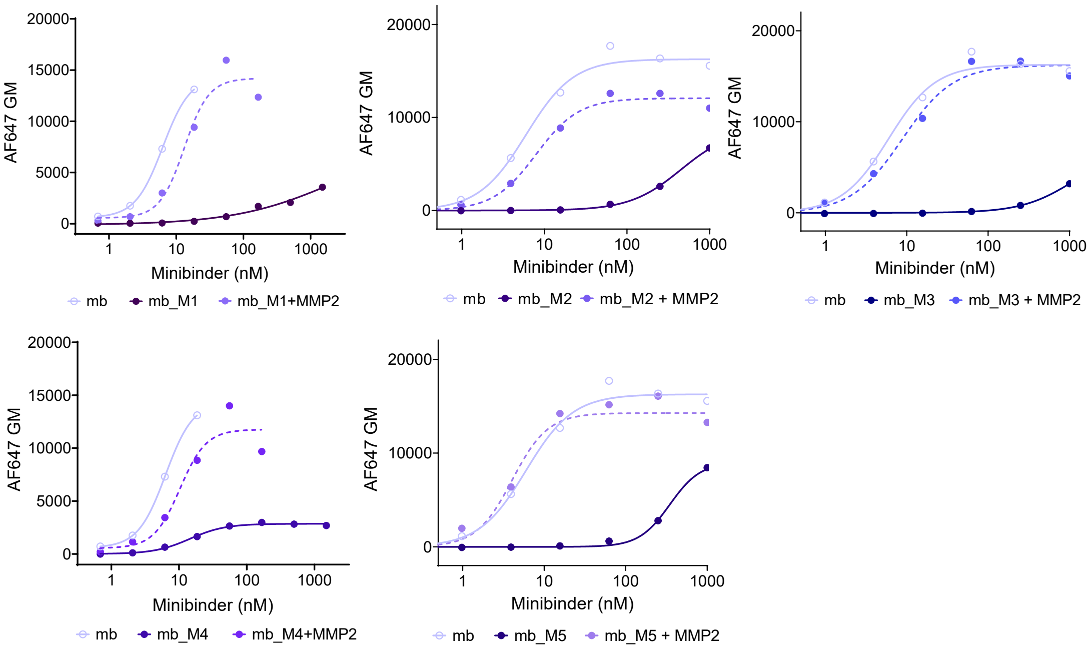

b

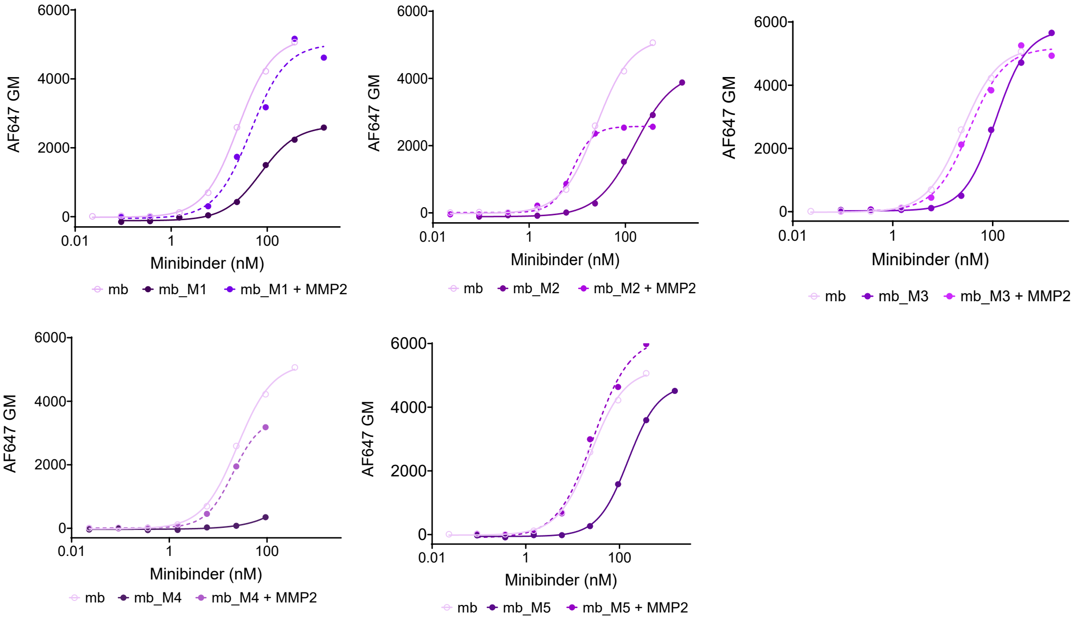

c

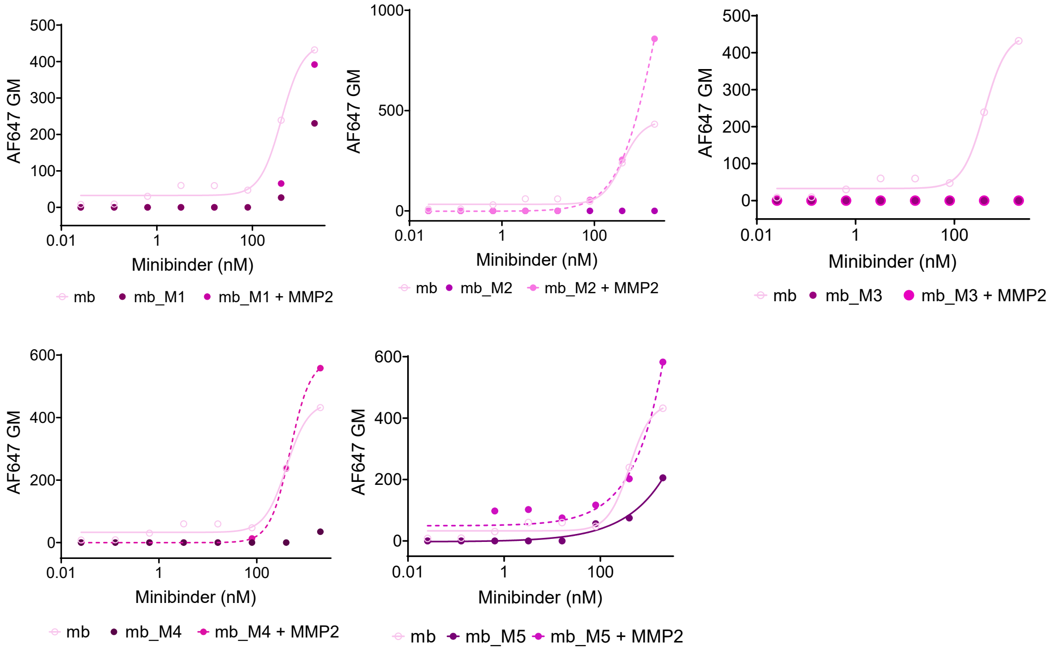

d

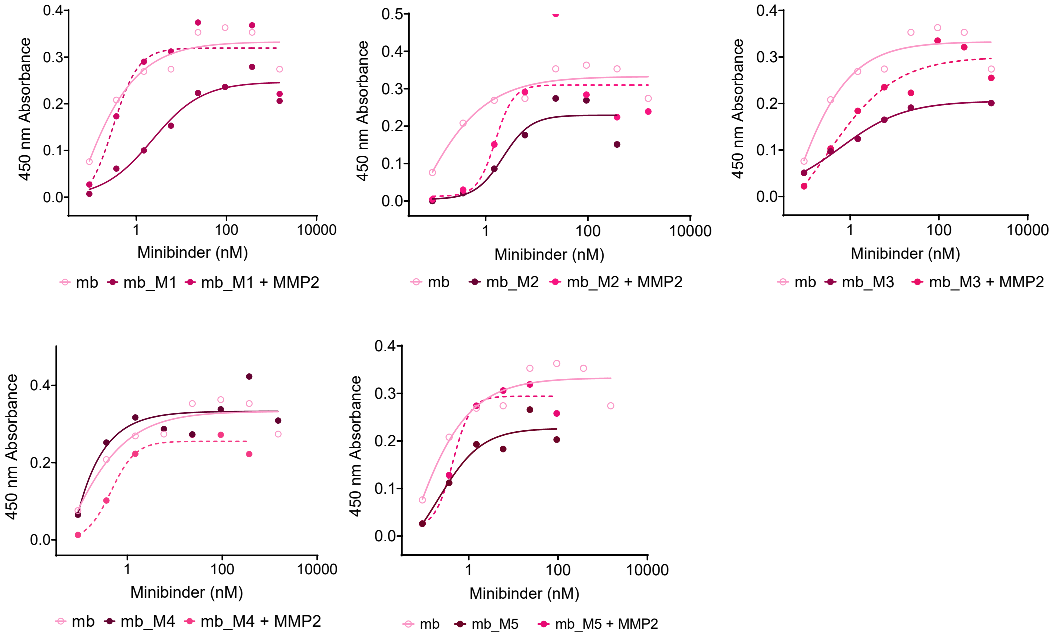

Figure S9. Binding curves of all expressed miniproteins by Flow Cytometry. Each graph shows the binding of the unmasked (mb), masked (M1-M5) and the recovered miniprotein after MMP-2 cleavage. Data of a) EGFRn, b) EGFRc, c) FGFR, and d) IL7Rα miniproteins is shown.

Further characterization of the lead candidate EGFRn mb_M3

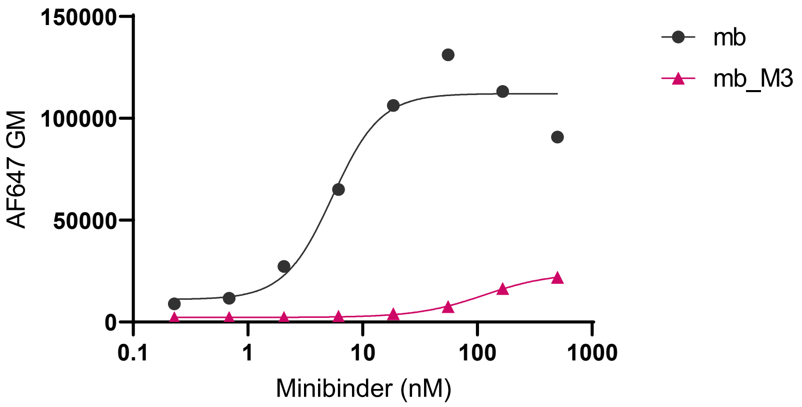

Figure S10. Binding curves of EGFRn mb_M3 at 37°C by Flow Cytometry.

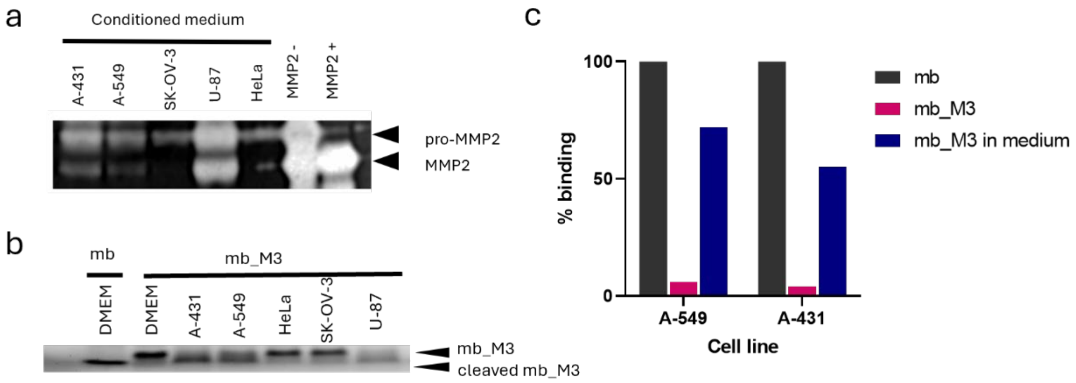

Figure S11. Cleavage of EGFRn mb_M3 with cell supernatant from different cell line cultures. a) Zymogram of cell supernatant after starvation; b) SDS-PAGE gel showing EGFRn mb_M3 after 16 h of cleavage in the cell supernatant of cell lines expressing different levels of MMP2; c) Binding of the mb_M3 (100 nM) after 16 h of cleavage in the cell supernatant of two cell lines.

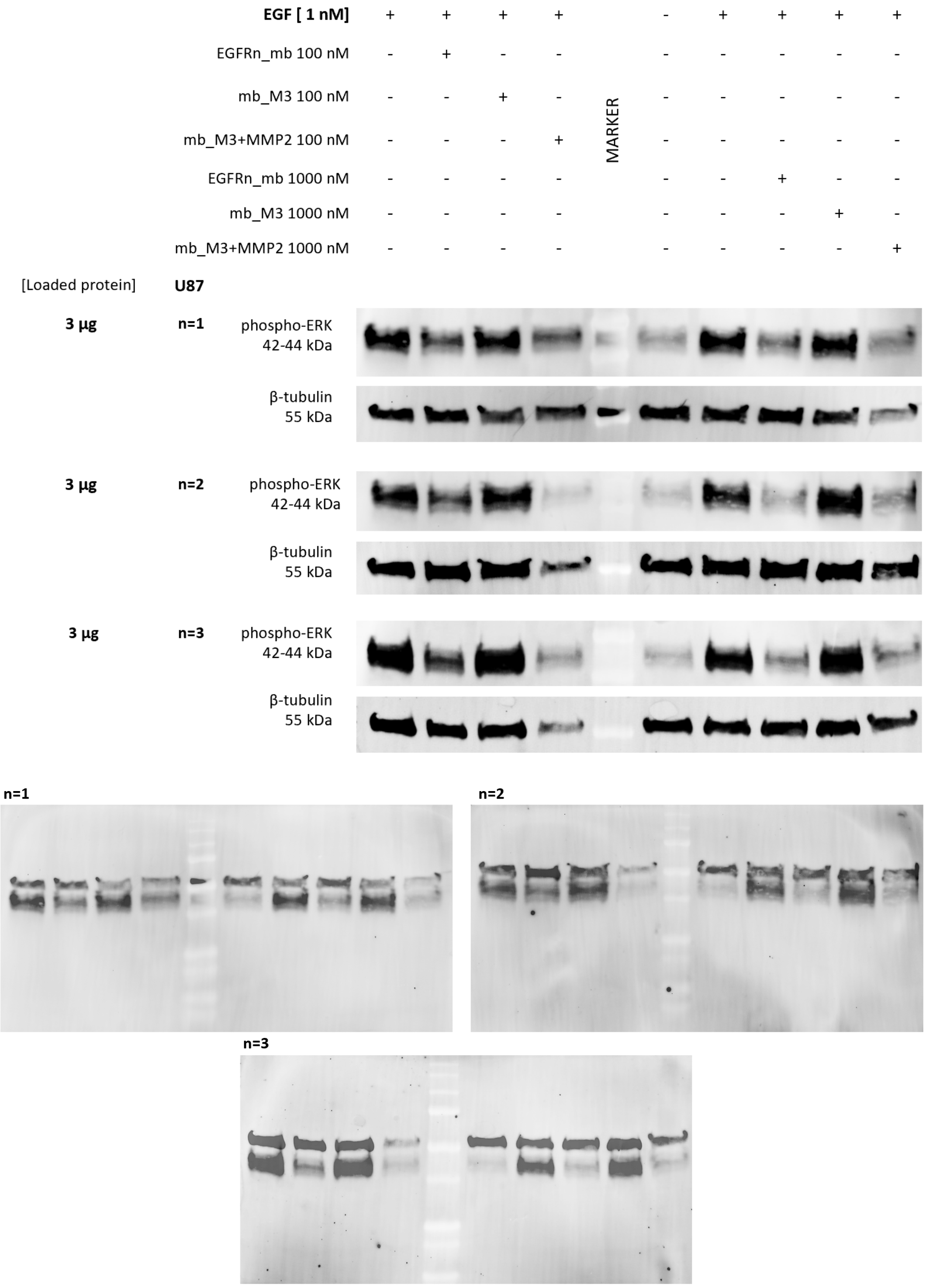

Figure S12a. Experimental characterization of the miniprotein binder effects on the native EGFR signaling. Western Blots were carried out at two different miniprotein binder concentrations: 100 nM and 1000 nM, respectively. n=3 biologically independent experimental repeats were performed which showed the antagonist effect of EGFRn_mb through the EGF-induced ERK phosphorylation reduction (lanes 2 and 8) The masking effect of mb_M3, in lanes 3 and 9, is lost upon proteolytic activation (lanes 4 and 10) proving the reversible inactivation with our mask design.

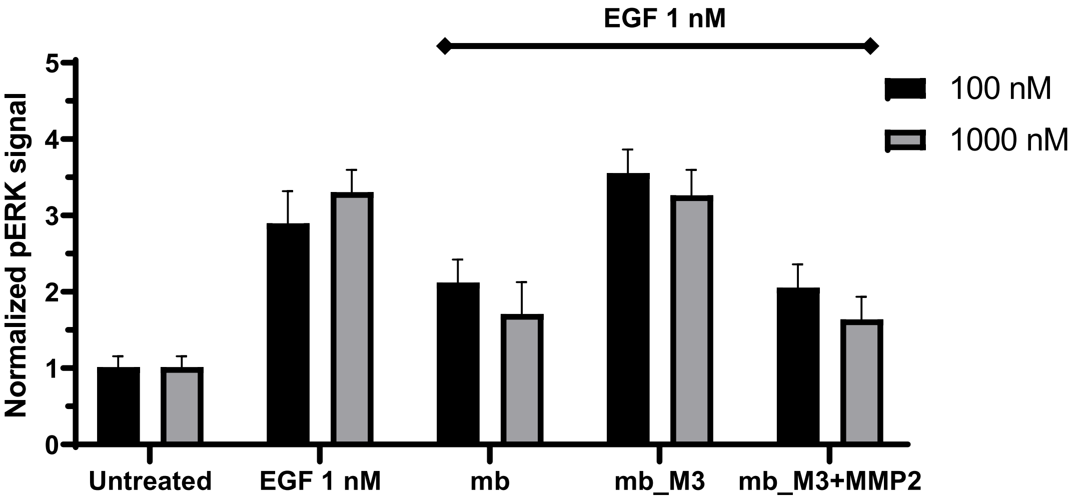

Figure S12b. Densitometric analysis of Western Blot membranes. Quantification was done using Image Lab software 6.1 considering n=3 biologically independent experimental repeats at two different concentrations of EGFRn_mb and mb_M3: 100 nM and 1000 nM.

EGFRn_mb

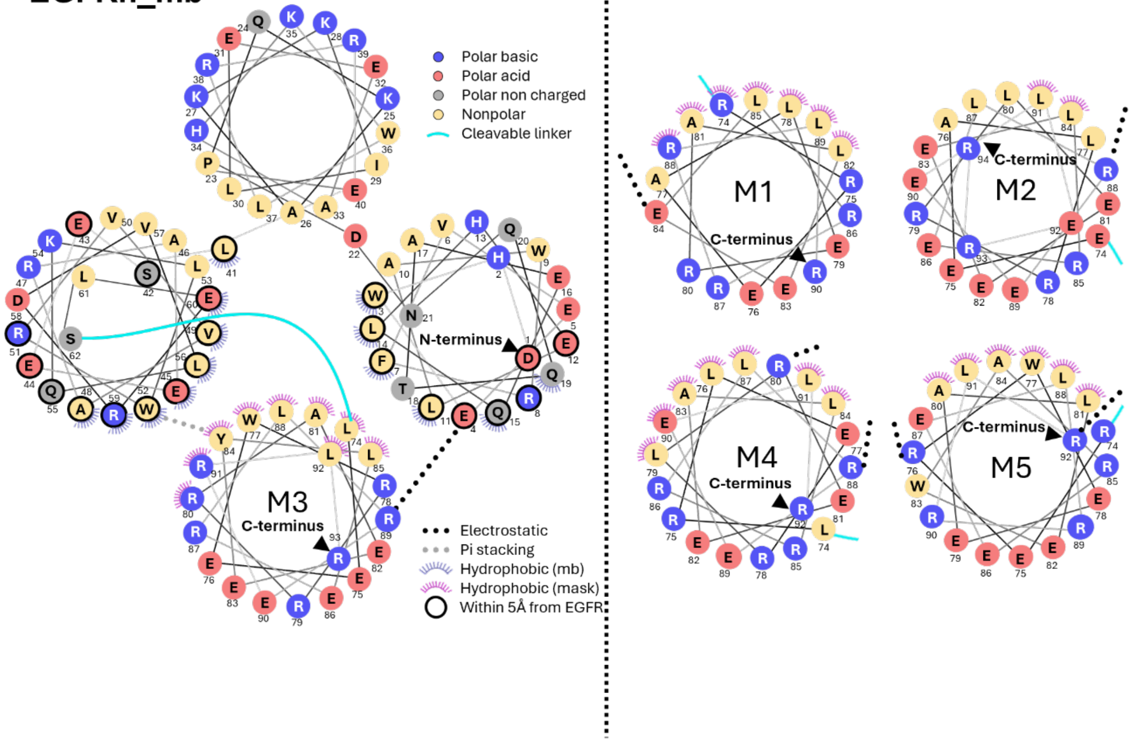

EGFRc_mb

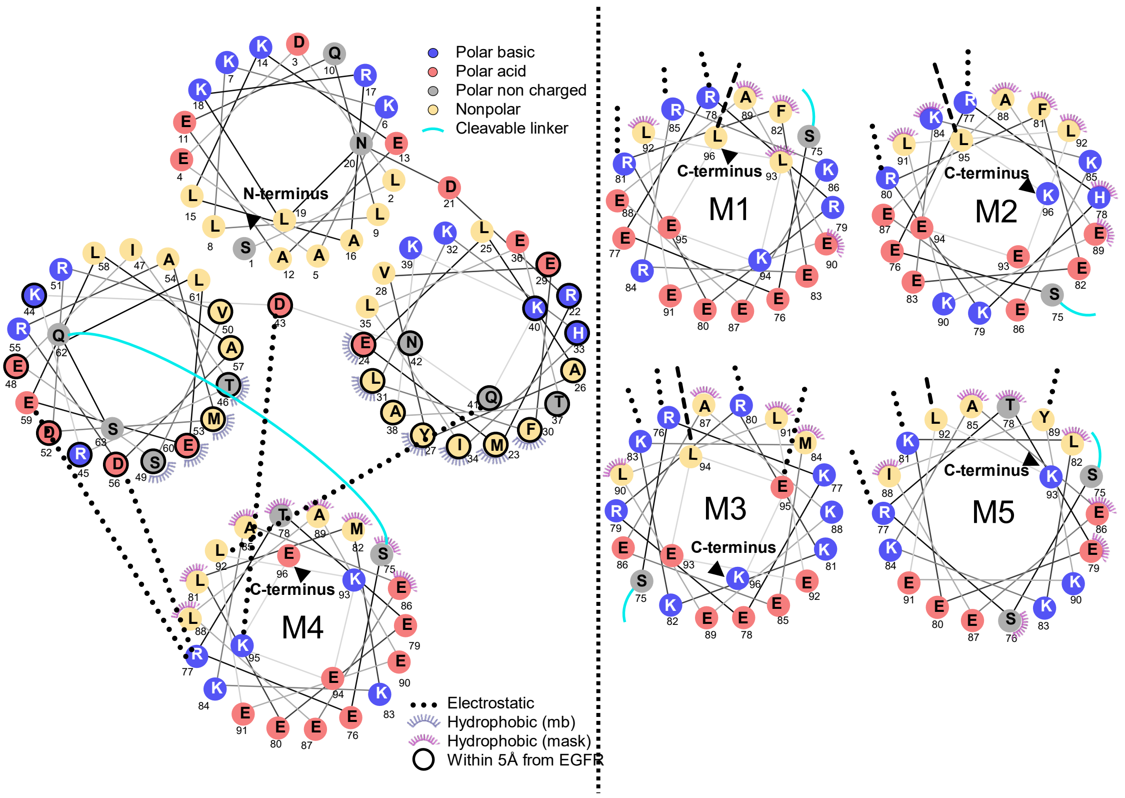

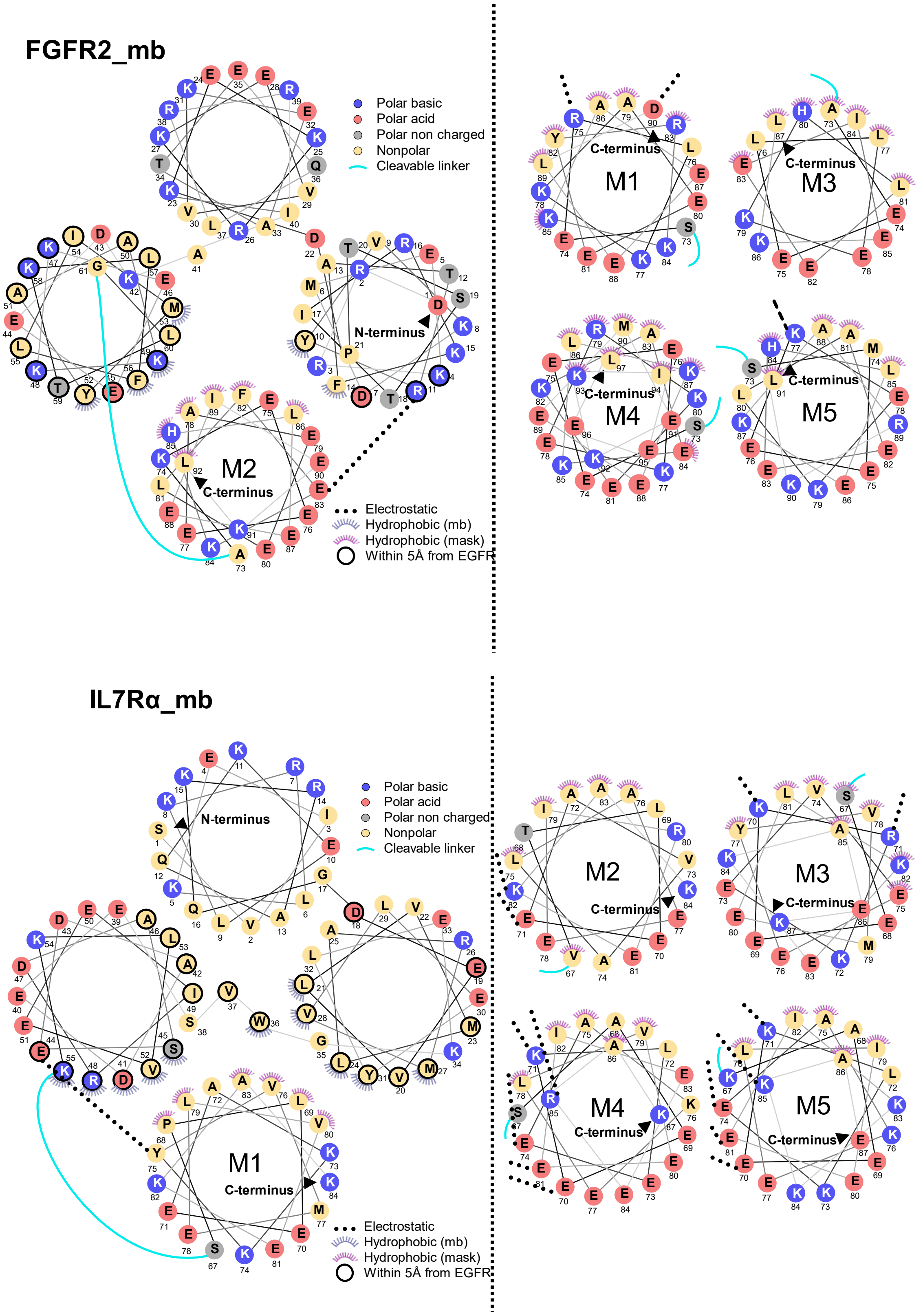

Figure S13. Helical wheel representation of EGFRn_mb, EGFRc_mb, FGFR2_mb and IL7Rα_mb with the best performing masks. The other masks are represented as separate helical wheels.

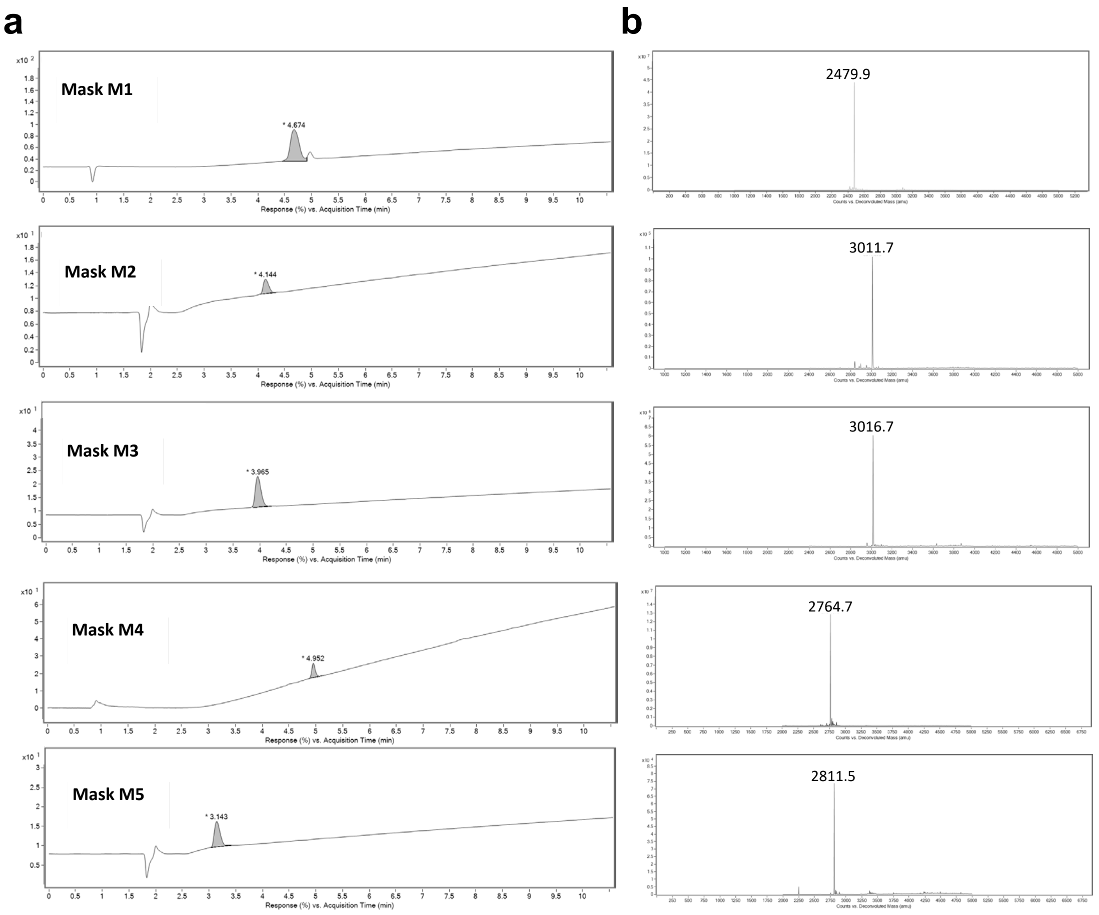

Figure S14. Characterization of synthetized masks. a) HPLC chromatogram and b) MS spectra of all EGFRn_mb masks synthesized by Solid-Phase Peptide Synthesis (SPPS).

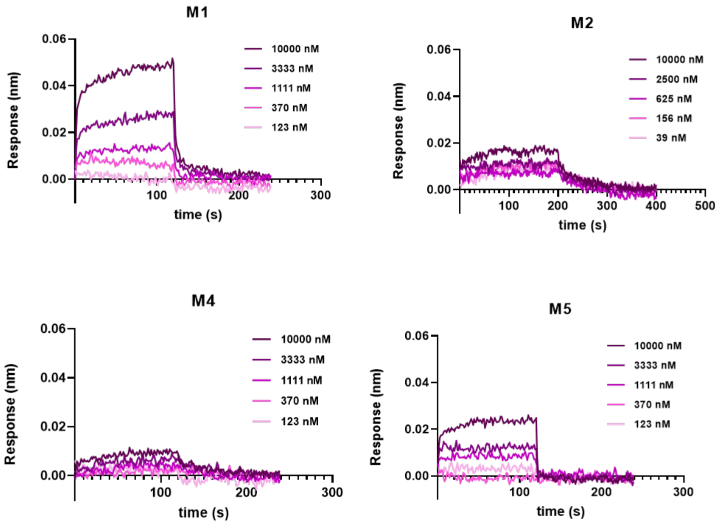

Figure S15. Biolayer Interferometry binding sensorgrams for peptidic masks vs. EGFRn_mb interactions. EGFRn_mb was first immobilized on biosensors followed by exposure to different concentrations of peptidic masks M1 to M5. M3 shows the highest affinity for the miniprotein.

Activation with different stimulus

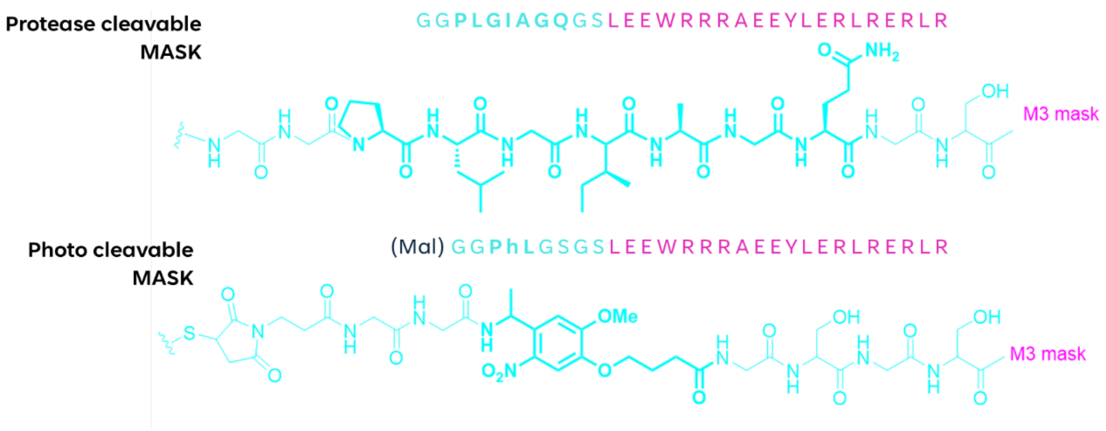

Figure S16. Comparison between protease-sensitive and photo-sensitive linkers used to tether the mask to the miniprotein binder.

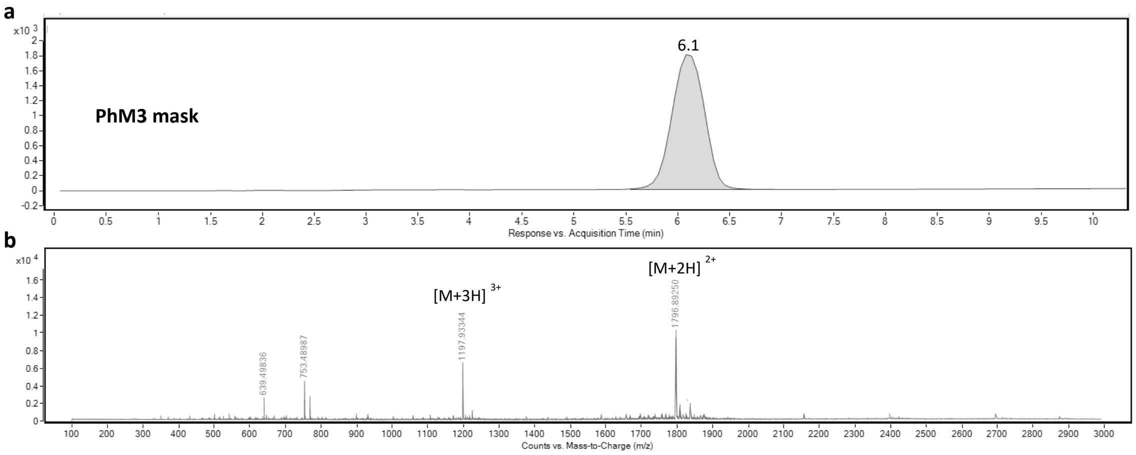

Figure S17. Characterization of PhM3 mask. a) HPLC chromatogram and b) MS spectra of PhM3 mask synthesized by Solid-Phase Peptide Synthesis (SPPS). Calculated MW: 3592.0 Da. Observed MW: 3591.4 Da

a

	Amino acid sequence
**mb**	MSGHHHHHHHHHHGSGDHWEEVFRWALEHLQEATQQNDPQKAKKILEEA HKWLRRELSEEEARAVVRWLKQLVDRELS
**mb_C**	MSGHHHHHHHHHHGSGDHWEEVFRWALEHLQEATQQNDPQKAKKILEEA HKWLRRELSEEEARAVVRWLKQLVDRELS**C**
**mb_CG (mb_Cys)**	MSGHHHHHHHHHHGSGDHWEEVFRWALEHLQEATQQNDPQKAKKILEEA HKWLRRELSEEEARAVVRWLKQLVDRELS**CG**

b

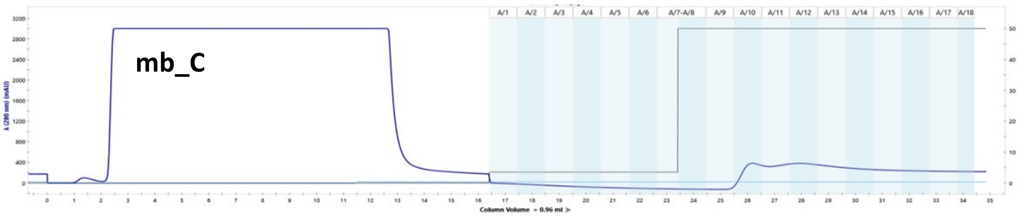

c

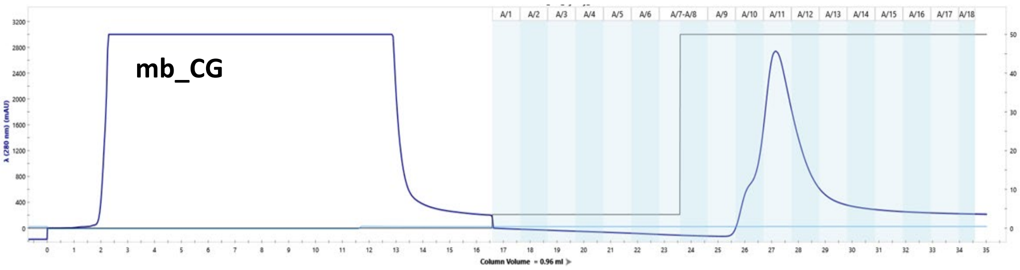

Figure S18. Constructs of miniproteins modified to encode an exposed Cysteine. a) Comparison of sequences of mb_C (ending with a Cysteine) and mb_CG (also named mb_Cys), which ends with Cysteine + Glycine. b-c) IMAC chromatograms of (b) mb_C and c) mb_CG.

a

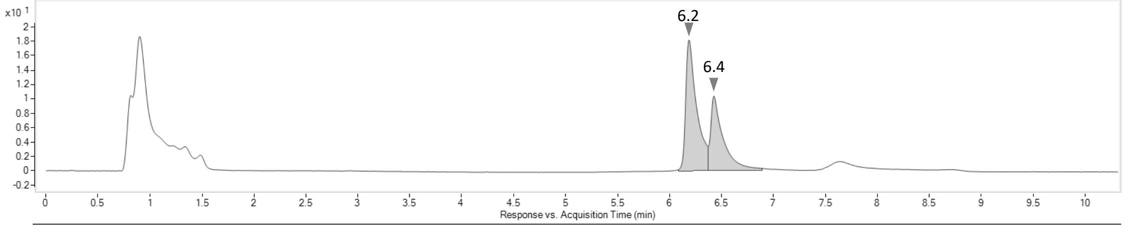

b

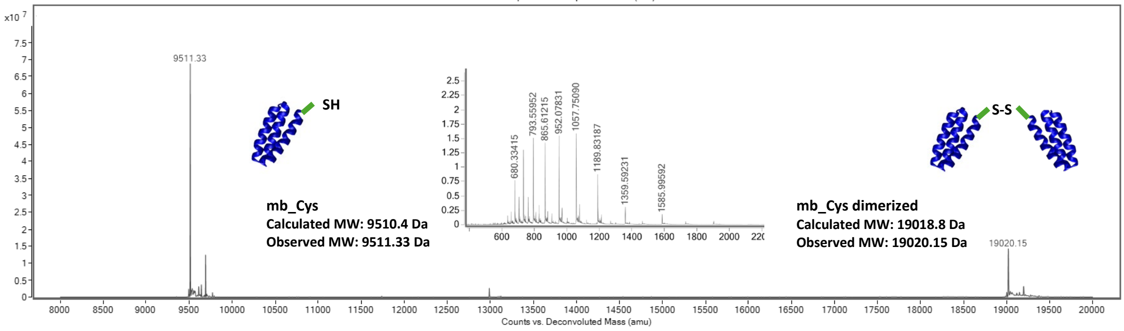

Figure S19. Characterization of mb_Cys after protein purification. a) HPLC chromatogram. Peak at retention time (rt) 6.2 min corresponds to the monomeric form, while peak at rt 6.4 min corresponds to the dimer. The applied gradient was 5-95% ACN over 10 min. b) MS spectrum.

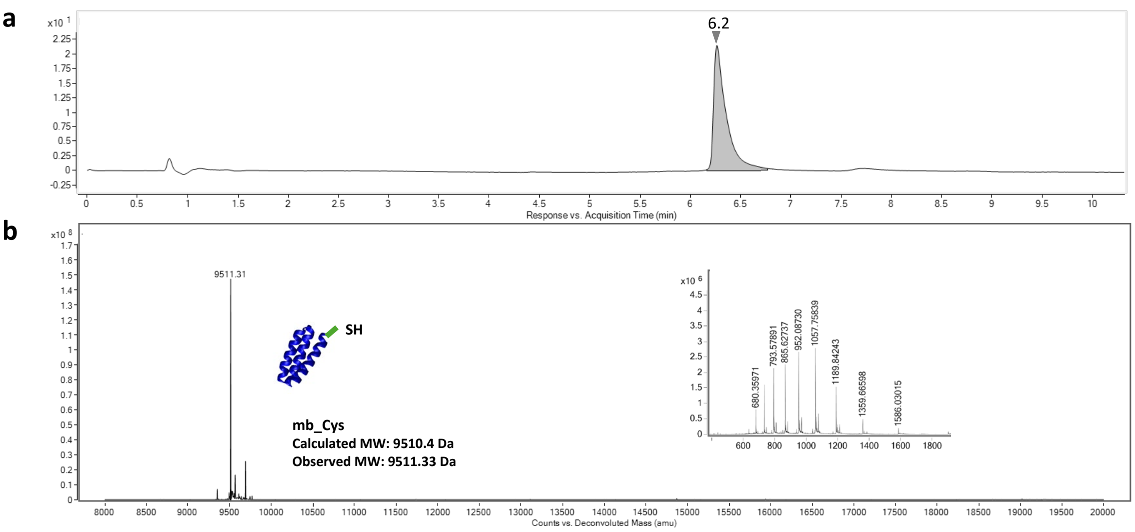

Figure S20. Characterization of mb_Cys after reduction with TCEP. a) HPLC chromatogram. The applied gradient was 5-95% ACN over 10 min. b) MS spectrum.

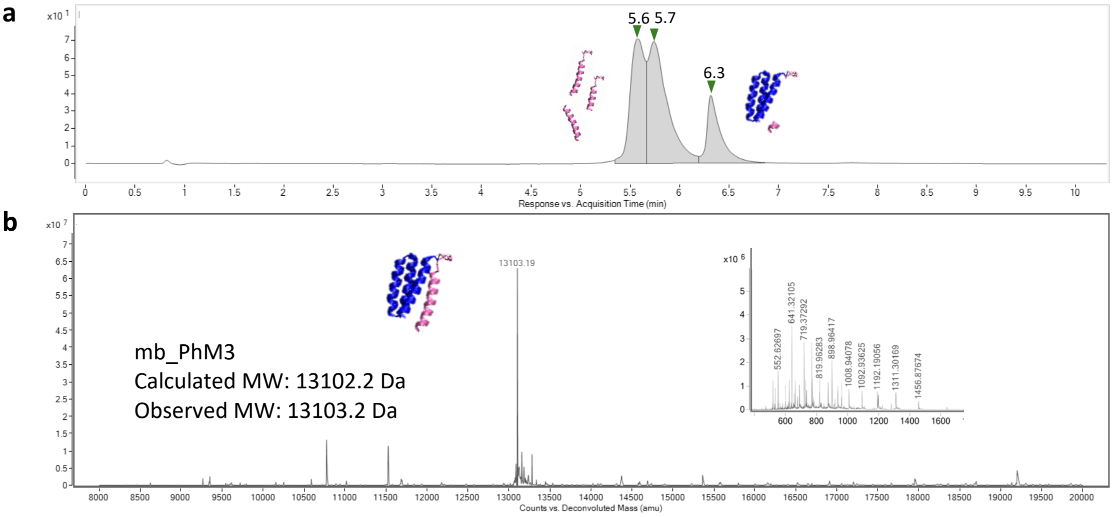

Figure S21. Conjugation of mb_Cys to mask to give PhM3 prior to purification. a) HPLC chromatogram. The applied gradient was 5-95% ACN over 10 min. Peaks at retention time 5.6 and 5.7 min correspond to excess peptide mask, and peak at 6.3 min corresponds to mb_PhM3. b) MS spectrum of mb_PhM3 conjugation, showing a main peak at 13103 Da, and the disappearance of the peak at 9511 Da.

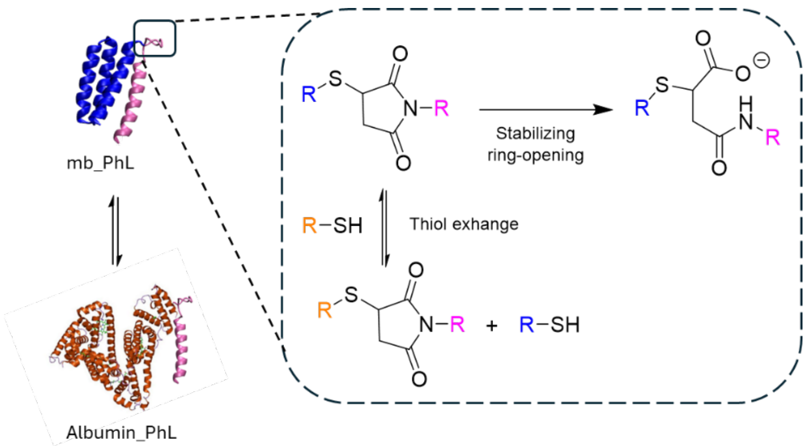

Figure S22. Schematic representation of maleimide ring opening, that prevents thiol exchange with other proteins as shown in Junutula *et al.*[50]

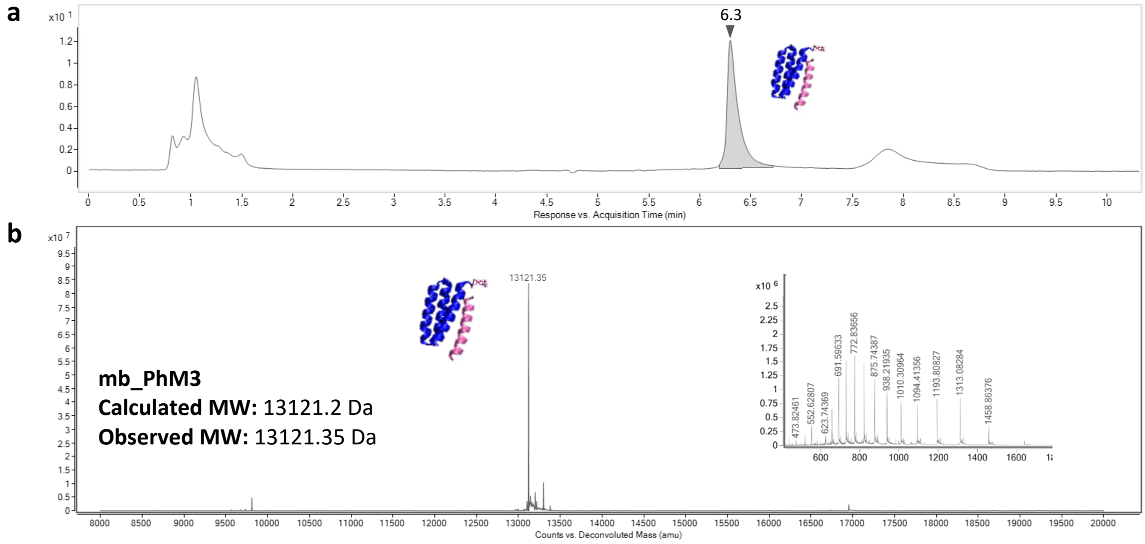

Figure S23. Characterization of purified mb_PhM3. a) HPLC chromatogram of purified mb_PhM3, using a gradient of 5-95% ACN over 10 min. b) MS spectrum of purified mb_PhM3.

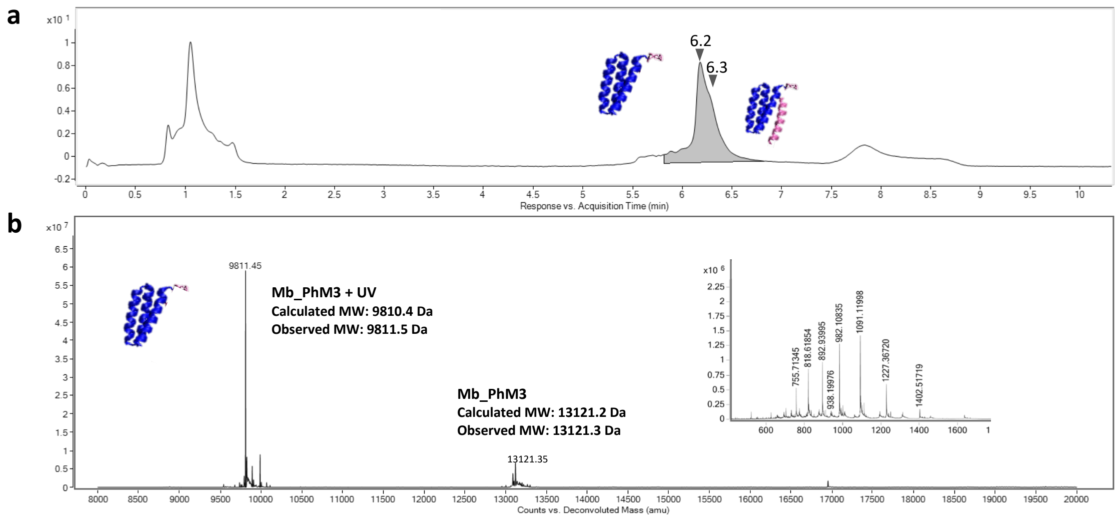

Figure S24. Characterization of the mb_PhM3 cleavage. a) HPLC chromatogram showing partially overlapped peaks at 6.2 min (non-cleaved mb_PhM3) and 6.3 min (cleaved mb_PhM3). The applied gradient was 5-95% ACN over 10 min. b) MS spectrum showing ca. 90% of mask cleavage.

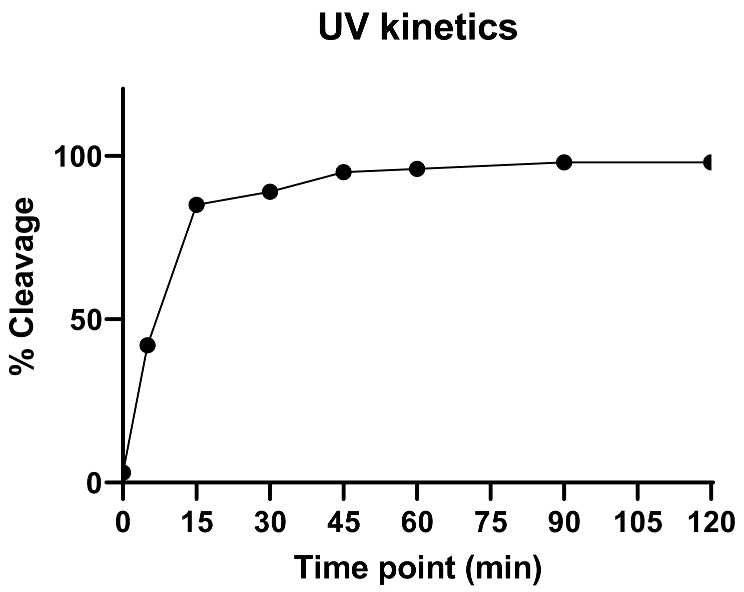

Figure S25. Cleavage kinetics of the light-sensitive M3_mb using UV light. M3_mb was exposed to 365 nm-light as in the experiments reported in this manuscript.

Supplementary Tables

Table S1. Gene blocks of expressed miniproteins.

		Gene blocks
**EGFRn_mb**	**mb**	*tttaactttaagaaggagatatacatatg*AGCGGTCACCATCATCATCATCACCATCAC<br>CATCACGGCAGCGGCGATCATTGGGAAGAAGTGTTTCGCtggGCGCT<br>GGAACATCTGCAGGAAGCGACCCAGCAGAACGATCCGCAGAAAGCC<br>AAGAAAATTCTGGAAGAGGCGCATAAGtggCTGCGCCGCGAACTGAG<br>CGAAGAAGAAGCGCGCgccGTGGTTCGTTGGCTGAAACAGCTGGTTGA<br>TCGCGAActgAGC*taactcgagtgagatccggctgctaacaaagcccgaaagg*
	**mb_M1**	*gctacat*ATGAGCGGTCACCATCATCATCATCACCATCACCATCACGGC<br>AGCGGCGATCATTGGGAAGAAGTGTTTCGCTGGGCGCTGGAACATC<br>TGCAGGAAGCGACCCAGCAGAACGATCCGCAGAAAGCCAAGAAAATT<br>CTGGAAGAGGCGCATAAGTGGCTGCGCCGCGAACTGAGCGAAGAA<br>GAAGCGCGCGCCGTGGTTCGTTGGCTGAAACAGCTGGTTGATCGCG<br>AACTGAGCGAGCCAGGGCCGCTGGGTATCGCAGGGCAAGGTTCTC<br>GCCGGGAGGCTTTAGAACGTGCCCTGGAGGAATTACGCCGTCGTCT<br>TCGT*taactcgagatcg*
	**mb_M2**	*gctacat*ATGAGCGGTCACCATCATCATCATCACCATCACCATCACGGC<br>AGCGGCGATCATTGGGAAGAAGTGTTTCGCTGGGCGCTGGAACATC<br>TGCAGGAAGCGACCCAGCAGAACGATCCGCAGAAAGCCAAGAAAATT<br>CTGGAAGAGGCGCATAAGTGGCTGCGCCGCGAACTGAGCGAAGAA<br>GAAGCGCGCGCCGTGGTTCGTTGGCTGAAACAGCTGGTTGATCGCG<br>AACTGAGCGGGTCAGGGGGGCCGCTTGGTATTGCAGGTCAGGGTTC<br>AGAAGAAGCCCTTCGGCGCTTAGAAGAGGAGCTGCGTGAGTTGCGG<br>GAGGAGTTAGAACGGCGC*taactcgagatcg*
	**mb_M3**	*gctacat*aTGAGCGGTCACCATCATCATCATCACCATCACCATCACGGCA<br>GCGGCGATCATTGGGAAGAAGTGTTTCGCTGGGCGCTGGAACATCT<br>GCAGGAAGCGACCCAGCAGAACGATCCGCAGAAAGCCAAGAAAATT<br>CTGGAAGAGGCGCATAAGTGGCTGCGCCGCGAACTGAGCGAAGAA<br>GAAGCGCGCGCCGTGGTTCGTTGGCTGAAACAGCTGGTTGATCGCG<br>AACTGAGCGGTGGGCCGCTGGGGATTGCGGGGCAGGGTTCCCTTGA<br>GGAGTGGCGTCGTCGGGCCGAAGAATACTTGGAACGGCTTCGTGAG<br>CGGTTACGT*taactcgagatcg*
	**mb_M4**	*gctacat*ATGAGCGGTCACCATCATCATCATCACCATCACCATCACGGC<br>AGCGGCGATCATTGGGAAGAAGTGTTTCGCTGGGCGCTGGAACATC<br>TGCAGGAAGCGACCCAGCAGAACGATCCGCAGAAAGCCAAGAAAATT<br>CTGGAAGAGGCGCATAAGTGGCTGCGCCGCGAACTGAGCGAAGAA<br>GAAGCGCGCGCCGTGGTTCGTTGGCTGAAACAGCTGGTTGATCGCG<br>AACTGAGCCAGCCTGGCCCGTTAGGTATCGCGGGGCAAGGCAGCTT<br>GCGTTTGGAGCGCCTCCGGGAGGAAGCGCTTCGTCGGCTTCGCGAA<br>GAACTTCGCtaactcgagatcg
	**mb_M5**	*gctacat*ATGAGCGGTCACCATCATCATCATCACCATCACCATCACGGC<br>AGCGGCGATCATTGGGAAGAAGTGTTTCGCTGGGCGCTGGAACATC<br>TGCAGGAAGCGACCCAGCAGAACGATCCGCAGAAAGCCAAGAAAATT<br>CTGGAAGAGGCGCATAAGTGGCTGCGCCGCGAACTGAGCGAAGAA<br>GAAGCGCGCGCCGTGGTTCGTTGGCTGAAACAGCTGGTTGATCGCG<br>AACTGAGCGAACCAGGCCCGTTGGGGATCGCCGGGCAGGGTAGCC<br>GGGAACGTTGGGAAGAAGCGCTTCGTGAAGCCCGGGAAGAGCTGCG<br>GCGCTTGCGG*taactcgagatcg*

Table S1. (continued)

	Gene blocks	
**EGFRc_mb**	**mb**	*ggtctctagga*CATCACCATCACCATCACGGCAGCGGCTCGTTAGATGAAGCAAAGAAACTACTGCAAGAGGCGGAGAAACTGGCACGTAAATTGAATGATCGGATGGAGCTGGCTTATGTGGAGTTCTTAAAGCATATCTTGGAGACGGCCAAAAAACAGAACGATAAACGAACCATTGAGAGTGTTCGTGACATGGCTCGTGACGCCCTTGAAGAACTCCAGTCC*taattccggagacc*
	**mb_M1**	*atcgggtctctagga*CATCACCATCACCATCACGGCAGCGGCTCGTTAGATGAAGCAAAGAAACTACTGCAAGAGGCGGAGAAACTGGCACGTAAATTGAATGATCGGATGGAGCTGGCTTATGTGGAGTTCTTAAAGCATATCTTGGAGACGGCCAAAAAACAGAACGATAAACGAACCATTGAGAGTGTTCGTGACATGGCTCGTGACGCCCTTGAAGAACTCCAGTCCGGCGGCCCGCTGGGCATTGCGGGCCAGGGCAGCAGCGAAGAACGCCGCGAACGCTTTGAACGCCGCAAAGAAGAAGCGGAAGAACTGCTGAAAGAACTG*taattccggagacctgca*
	**mb_M2**	*atcgggtctctagga*CATCACCATCACCATCACGGCAGCGGCTCGTTAGATGAAGCAAAGAAACTACTGCAAGAGGCGGAGAAACTGGCACGTAAATTGAATGATCGGATGGAGCTGGCTTATGTGGAGTTCTTAAAGCATATCTTGGAGACGGCCAAAAAACAGAACGATAAACGAACCATTGAGAGTGTTCGTGACATGGCTCGTGACGCCCTTGAAGAACTCCAGTCCGGCGGCCCGCTGGGCATTGCGGGCCAGGGCAGCAGCGAACGCCATAAACGCTTTGAAGAAAAAAAAGAAGAAGCGGAAAAACTGCTGGAAGAACTGAAA*taattccggagacctgca*
	**mb_M3**	*atcgggtctctagga*CATCACCATCACCATCACGGCAGCGGCTCGTTAGATGAAGCAAAGAAACTACTGCAAGAGGCGGAGAAACTGGCACGTAAATTGAATGATCGGATGGAGCTGGCTTATGTGGAGTTCTTAAAGCATATCTTGGAGACGGCCAAAAAACAGAACGATAAACGAACCATTGAGAGTGTTCGTGACATGGCTCGTGACGCCCTTGAAGAACTCCAGTCCGGCGGCCCGCTGGGCATTGCGGGCCAGGGCAGCAGCCGGAAAGAACGTCGCAAAAAGAAAATGGAAGAAGCCAAAGAACTGTTGGAAGAACTGGAGAAA*taattccggagacctgca*
	**mb_M4**	*atcgggtctctagga*CATCACCATCACCATCACGGCAGCGGCTCGTTAGATGAAGCAAAGAAACTACTGCAAGAGGCGGAGAAACTGGCACGTAAATTGAATGATCGGATGGAGCTGGCTTATGTGGAGTTCTTAAAGCATATCTTGGAGACGGCCAAAAAACAGAACGATAAACGAACCATTGAGAGTGTTCGTGACATGGCTCGTGACGCCCTTGAAGAACTCCAGTCCGGCGGCCCGCTGGGCATTGCGGGCCAGGGCAGCTCAGAACGCACGGAGGAGCTGATGAAAAAGGCAGAAGAACTGGCCGAAGAACTAAAAGAGAAAGAG*taattccggagacctgca*
	**mb_M5**	*atcgggtctctagga*CATCACCATCACCATCACGGCAGCGGCTCGTTAGATGAAGCAAAGAAACTACTGCAAGAGGCGGAGAAACTGGCACGTAAATTGAATGATCGGATGGAGCTGGCTTATGTGGAGTTCTTAAAGCATATCTTGGAGACGGCCAAAAAACAGAACGATAAACGAACCATTGAGAGTGTTCGTGACATGGCTCGTGACGCCCTTGAAGAACTCCAGTCCGGCGGCCCGCTGGGCATTGCGGGCCAGGGCAGCTCCTCACGTACCGAAGAAAAACTGAAAAAAGCGGAAGAAATTTATAAAGAACTGAAA*taattccggagacctgca*

Table S1. (continued)

	Gene blocks	
**FGFR2_mb**	**mb**	*atcgggtctctagga*CATCACCATCACCATCACGGCAGCGGCGATCGCCGCAAGGAAATGGATAAAGTGTATCGTACCGCATTTAAACGGATCACAAGCACGCCAGACAAGGAGAAACGAAAAGAAGTTGTCAAAGAAGCCACCGAGCAGCTCAGACGTATCGCGAAGGACGAAGAAGAGAAGAAAAAAGCGGCTTACATGATTCTGTTCCTGAAAACTTTAGGA*taattccggagacctgca*
	**mb_M1**	*atcgggtctctagga*CATCACCATCACCATCACGGCAGCGGCGATCGCCGCAAGGAAATGGATAAAGTGTATCGTACCGCATTTAAACGGATCACAAGCACGCCAGACAAGGAGAAACGAAAAGAAGTTGTCAAAGAAGCCACCGAGCAGCTCAGACGTATCGCGAAGGACGAAGAAGAGAAGAAAAAAGCGGCTTACATGATTCTGTTCCTGAAAACTTTAGGAGGTGGCCCGCTTGGTATTGCGGGCCAAGGGTCCAGTGAGCGCTTGAAAAAAGCCGAAGAATATCGTAAAAAAGCAGAAGAACTGGAT*taattccggagacctgca*
	**mb_M2**	*atcgggtctctagga*CATCACCATCACCATCACGGCAGCGGCGATCGCCGCAAGGAAATGGATAAAGTGTATCGTACCGCATTTAAACGGATCACAAGCACGCCAGACAAGGAGAAACGAAAAGAAGTTGTCAAAGAAGCCACCGAGCAGCTCAGACGTATCGCGAAGGACGAAGAAGAGAAGAAAAAAGCGGCTTACATGATTCTGTTCCTGAAAACTTTAGGAGGTGGCCCGCTTGGTATTGCGGGCCAAGGGTCCGCAAAAGAAGAGGAAGCGGAAGAACTGTTTGAAAAACATCTGGAGGAGATTGAAAAACTG*taattccggagacctgca*
	**mb_M3**	*atcgggtctctagga*CATCACCATCACCATCACGGCAGCGGCGATCGCCGCAAGGAAATGGATAAAGTGTATCGTACCGCATTTAAACGGATCACAAGCACGCCAGACAAGGAGAAACGAAAAGAAGTTGTCAAAGAAGCCACCGAGCAGCTCAGACGTATCGCGAAGGACGAAGAAGAGAAGAAAAAAGCGGCTTACATGATTCTGTTCCTGAAAACTTTAGGAGGTGGCCCGCTTGGTATTGCGGGCCAAGGGTCCGCCGAAGAGTTGCTGGAAAAACATCTTGAAGAGATCGAAAAGTTA*taattccggagacctgca*
	**mb_M4**	*atcgggtctctagga*CATCACCATCACCATCACGGCAGCGGCGATCGCCGCAAGGAAATGGATAAAGTGTATCGTACCGCATTTAAACGGATCACAAGCACGCCAGACAAGGAGAAACGAAAAGAAGTTGTCAAAGAAGCCACCGAGCAGCTCAGACGTATCGCGAAGGACGAAGAAGAGAAGAAAAAAGCGGCTTACATGATTCTGTTCCTGAAAACTTTAGGAGGTGGCCCGCTTGGTATTGCGGGCCAAGGGTCCAGCGAAGAAGAAAAGGAGCGTAAAGAGAAGGCCGAGAAGCTCAAGGAGGAAATGGAGAAAAAAATTGAAGAATTG*taattccggagacctgca*
	**mb_M5**	*atcgggtctctagga*CATCACCATCACCATCACGGCAGCGGCGATCGCCGCAAGGAAATGGATAAAGTGTATCGTACCGCATTTAAACGGATCACAAGCACGCCAGACAAGGAGAAACGAAAAGAAGTTGTCAAAGAAGCCACCGAGCAGCTCAGACGTATCGCGAAGGACGAAGAAGAGAAGAAAAAAGCGGCTTACATGATTCTGTTCCTGAAAACTTTAGGAGGTGGCCCGCTTGGTATTGCGGGCCAAGGGTCCAGCATGGAGGAGAAAGAAAAACTGGCAGAAGAACATCTGGAAAAAGCACGCAAACTG*taattccggagacctgca*

Table S1. (continued)

	Gene blocks	
**IL7Rα_mb**	**mb**	*atcgggtctctagga*CATCACCATCACCATCACGGCAGCGGCTCCGTG<br>ATTGAAAAACTGCGTAAACTTGAAAAACAAGCGCGCAAACAGG<br>GTGACGAAGTCTTAGTCATGTTGGCCCGGATGGTACTGGAAT<br>ACCTCGAAAAAGGCTGGGTTTCAGAGGAAGATGCGGATGAGA<br>GCGCAGATCGCATTGAAGAGGTGCTGAAGAAA*taattccggagacctgca*
	**mb_M1**	*atcgggtctctagga*CATCACCATCACCATCACGGCAGCGGCTCCGTG<br>ATTGAAAAACTGCGTAAACTTGAAAAACAAGCGCGCAAACAGG<br>GTGACGAAGTCTTAGTCATGTTGGCCCGGATGGTACTGGAAT<br>ACCTCGAAAAAGGCTGGGTTTCAGAGGAAGATGCGGATGAGA<br>GCGCAGATCGCATTGAAGAGGTGCTGAAGAAAGGGGGTCCAC<br>TAGGCATCGCCGGCCAGGGATCGAGTCCGCTGGAGGAAGCT<br>AAGAAATATGTTATGGAACTGGTGGAAAAGGCAAAA*taattccggaga*<br>*cctgca*
	**mb_M2**	*atcgggtctctagga*CATCACCATCACCATCACGGCAGCGGCTCCGTG<br>ATTGAAAAACTGCGTAAACTTGAAAAACAAGCGCGCAAACAGG<br>GTGACGAAGTCTTAGTCATGTTGGCCCGGATGGTACTGGAAT<br>ACCTCGAAAAAGGCTGGGTTTCAGAGGAAGATGCGGATGAGA<br>GCGCAGATCGCATTGAAGAGGTGCTGAAGAAAGGGGGTCCAC<br>TAGGCATCGCCGGCCAGGGATCGGTCACCCTTGAAGAGGCC<br>GTTGCACTGGCGGAGGAAATTCGTGAGAAAGCGAAA*taattccggaga*<br>*cctgca*
	**mb_M3**	*atcgggtctctagga*CATCACCATCACCATCACGGCAGCGGCTCCGTG<br>ATTGAAAAACTGCGTAAACTTGAAAAACAAGCGCGCAAACAGG<br>GTGACGAAGTCTTAGTCATGTTGGCCCGGATGGTACTGGAAT<br>ACCTCGAAAAAGGCTGGGTTTCAGAGGAAGATGCGGATGAGA<br>GCGCAGATCGCATTGAAGAGGTGCTGAAGAAAGGGGGTCCAC<br>TAGGCATCGCCGGCCAGGGATCGTCGGAGGAAAAACGCAAAG<br>AGGTAGAAGAGTACGTCATGGAATTGAAAGAGAAAGCAGAAAA<br>G*taattccggagacctgca*
	**mb_M4**	*atcgggtctctagga*CATCACCATCACCATCACGGCAGCGGCTCCGTG<br>ATTGAAAAACTGCGTAAACTTGAAAAACAAGCGCGCAAACAGG<br>GTGACGAAGTCTTAGTCATGTTGGCCCGGATGGTACTGGAAT<br>ACCTCGAAAAAGGCTGGGTTTCAGAGGAAGATGCGGATGAGA<br>GCGCAGATCGCATTGAAGAGGTGCTGAAGAAAGGGGGTCCAC<br>TAGGCATCGCCGGCCAGGGATCGTCAGCAGAAGAAAAACTGG<br>AGGAAGCGAAAGAACTAGTGGAAGAAATCGAAGAACGGGCCA<br>AA*taattccggagacctgca*
	**mb_M5**	*atcgggtctctagga*CATCACCATCACCATCACGGCAGCGGCTCCGTG<br>ATTGAAAAACTGCGTAAACTTGAAAAACAAGCGCGCAAACAGG<br>GTGACGAAGTCTTAGTCATGTTGGCCCGGATGGTACTGGAAT<br>ACCTCGAAAAAGGCTGGGTTTCAGAGGAAGATGCGGATGAGA<br>GCGCAGATCGCATTGAAGAGGTGCTGAAGAAAGGGGGTCCAC<br>TAGGCATCGCCGGCCAGGGATCGAAAGCCGAGGAAAAATTGA<br>AGGAAGCGAAAGAACTGATCGAAGAAATTAAAAAAAAAGCAGA<br>A*taattccggagacctgca*

Table S2. Amino acid sequences of expressed miniproteins.

		Amino acid sequences of miniproteins
**EGFRn_mb**	**mb_M1**	MSGHHHHHHHHHHGSGDHWEEVFRWALEHLQEATQQNDPQKAKKILEEAHK<br>WLRRELSEEEARAVVRWLKQLVDRELSEPGPLGIAGQGSRREALERALEELRRRLR
	**mb_M2**	MSGHHHHHHHHHHGSGDHWEEVFRWALEHLQEATQQNDPQKAKKILEEAHK<br>WLRRELSEEEARAVVRWLKQLVDRELSGSGGPLGIAGQGSEEALRRLEEELRELRE<br>ELERR
	**mb_M3**	MSGHHHHHHHHHHGSGDHWEEVFRWALEHLQEATQQNDPQKAKKILEEAHK<br>WLRRELSEEEARAVVRWLKQLVDRELSGGPLGIAGQGSLEEWRRRAEEYLERLRE<br>RLR
	**mb_M4**	MSGHHHHHHHHHHGSGDHWEEVFRWALEHLQEATQQNDPQKAKKILEEAHK<br>WLRRELSEEEARAVVRWLKQLVDRELSQPGPLGIAGQGSLRLERLREEALRRLREE<br>LR
	**mb_M5**	MSGHHHHHHHHHHGSGDHWEEVFRWALEHLQEATQQNDPQKAKKILEEAHK<br>WLRRELSEEEARAVVRWLKQLVDRELSEPGPLGIAGQGSRERWEEALREAREELR<br>RLR
**EGFRc_mb**	**mb_M1**	MSSGHHHHHHGSGSLDEAKKLLQEAEKLARKLNDRMELAYVEFLKHILETAKKQ<br>NDKRTIESVRDMARDALEELQSGGPLGIAGQGSSEERRERFERRKEEAEELLKEL
	**mb_M2**	MSSGHHHHHHGSGSLDEAKKLLQEAEKLARKLNDRMELAYVEFLKHILETAKKQ<br>NDKRTIESVRDMARDALEELQSGGPLGIAGQGSSERHKRFEEKKEEAEKLLEELK
	**mb_M3**	MSSGHHHHHHGSGSLDEAKKLLQEAEKLARKLNDRMELAYVEFLKHILETAKKQ<br>NDKRTIESVRDMARDALEELQSGGPLGIAGQGSSRKERRKKKMEEAKELLEELEK
	**mb_M4**	MSSGHHHHHHGSGSLDEAKKLLQEAEKLARKLNDRMELAYVEFLKHILETAKKQ<br>NDKRTIESVRDMARDALEELQSGGPLGIAGQGSSERTEELMKKAEELAEELKEKE
	**mb_M5**	MSSGHHHHHHGSGSLDEAKKLLQEAEKLARKLNDRMELAYVEFLKHILETAKKQ<br>NDKRTIESVRDMARDALEELQSGGPLGIAGQGSSSRTEEKLKKAEEIYKELK
**FGFR2_mb**	**mb_M1**	MSSGHHHHHHGSGDRRKEMDKVYRTAFKRITSTPDKEKRKEVVKEATEQLRRIA<br>KDEEEKKKAAYMILFLKTLGGGPLGIAGQGSSERLKKAEEYRKKAEELD
	**mb_M2**	MSSGHHHHHHGSGDRRKEMDKVYRTAFKRITSTPDKEKRKEVVKEATEQLRRIA<br>KDEEEKKKAAYMILFLKTLGGGPLGIAGQGSAKEEEAEELFEKHLEEIEKL
	**mb_M3**	MSSGHHHHHHGSGDRRKEMDKVYRTAFKRITSTPDKEKRKEVVKEATEQLRRIA<br>KDEEEKKKAAYMILFLKTLGGGPLGIAGQGSAEELLEKHLEEIEKL
	**mb_M4**	MSSGHHHHHHGSGDRRKEMDKVYRTAFKRITSTPDKEKRKEVVKEATEQLRRIA<br>KDEEEKKKAAYMILFLKTLGGGPLGIAGQGSSEEEKERKEKAEKLKEEMEKKIEEL
	**mb_M5**	MSSGHHHHHHGSGDRRKEMDKVYRTAFKRITSTPDKEKRKEVVKEATEQLRRIA<br>KDEEEKKKAAYMILFLKTLGGGPLGIAGQGSSMEEKEKLAEEHLEKARKL
**IL7Rα_mb**	**mb_M1**	MSSGHHHHHHGSGSVIEKLRKLEKQARKQGDEVLVMLARMVLEYLEKGWVSEE<br>DADESADRIEEVLKKGGPLGIAGQGSSPLEEAKKYVMELVEKAK
	**mb_M2**	MSSGHHHHHHGSGSVIEKLRKLEKQARKQGDEVLVMLARMVLEYLEKGWVSEE<br>DADESADRIEEVLKKGGPLGIAGQGSVTLEEAVALAEEIREKAK
	**mb_M3**	MSSGHHHHHHGSGSVIEKLRKLEKQARKQGDEVLVMLARMVLEYLEKGWVSEE<br>DADESADRIEEVLKKGGPLGIAGQGSSEEKRKEVEEYVMELKEKAEK
	**mb_M4**	MSSGHHHHHHGSGSVIEKLRKLEKQARKQGDEVLVMLARMVLEYLEKGWVSEE<br>DADESADRIEEVLKKGGPLGIAGQGSSAEEKLEEAKELVEEIEERAK
	**mb_M5**	MSSGHHHHHHGSGSVIEKLRKLEKQARKQGDEVLVMLARMVLEYLEKGWVSEE<br>DADESADRIEEVLKKGGPLGIAGQGSKAEEKLKEAKELIEEIKKKAE

Table S3. Calculated and observed Molecular Weights (MW) of expressed unmasked (mb), masked (M) and MMP-2 cleaved or recovered miniproteins.

		Masked		MMP-2 cleaved (recovered)	
Minibinder (mb)	Mask (M)	Calculated MW [Da]	Observed MW [Da]	Calculated MW [Da]	Observed MW [Da]
**EGFRn_mb**	mb	9 350.2	9 350.7	9 350.7	9 350.7
	M1	12 619.0	12 619.5	9 900.8	9 901.3
	M2	13 125.4	13 125.8	9875.8	9 976.4
	M3	12 986.3	12 986.7	9 731.7	9 732.3
	M4	12 902.4	12 902.7	9 899.9	9 900.4
	M5	12 950.3	12 950.7	9 900.9	9 901.4
**EGFRc_mb**	mb	8 656.7	8 657.3	8 657.3	8 657.3
	M1	12 395.7	12 396.5	9 038.1	9 038.7
	M2	12 319.7	12 320.4	9 038.1	9 038.6
	M3	12 321.9	12 322.5	9 038.1	9 038.8
	M4	12 183.5	12 184.3	9 038.1	9 038.6
	M5	11 843.2	11 843.9	9 038.1	9 038.8
**FGFR2_mb**	mb	8 600.8	8 601.4	8 601.4	8 601.4
	M1	11 700.2	11 700.8	8 982.2	8 982.5
	M2	11 921.4	11 922.2	8 982.2	8 982.6
	M3	11 300.8	11 3001.5	8 982.2	8 982.6
	M4	12 598.3	12 598.9	8 982.2	8 982.6
	M5	11 776.4	11 776.9	8 982.2	8 982.6
**IL7Rα_mb**	mb	7 687.6	7 688.2	7 688.2	7 688.2
	M1	10 657.1	10 657.6	8 069.5	8 069.7
	M2	10 563.9	10 564.5	8 069.5	8 069.6
	M3	11 176.5	11 177.2	8 069.5	8 069.6
	M4	11 024.3	11 024.8	8 069.5	8 069.6
	M5	11049.5	11050.2	8 069.5	8 069.6

Table S4. Affinity of parental miniproteins.

Minibinder (mb)	K_D reported [27] (BLI) [nM]	Measured IC50 [nM]	Assessed in
**EGFRn_mb**	1.2	7	A-431
**EGFRc_mb**	6.8	25	A-431
**FGFR2_mb**	243	406	SK-BR-3
**IL7Rα_mb**	0.3	0.4	ELISA

References

(21) Watson, J. L.; Juergens, D.; Bennett, N. R.; Trippe, B. L.; Yim, J.; Eisenach, H. E.; Ahern, W.; Borst, A. J.; Ragotte, R. J.; Milles, L. F.; Wicky, B. I. M.; Hanikel, N.; Pellock, S. J.; Courbet, A.; Sheffler, W.; Wang, J.; Venkatesh, P.; Sappington, I.; Torres, S. V.; Lauko, A.; De Bortoli, V.; Mathieu, E.; Ovchinnikov, S.; Barzilay, R.; Jaakkola, T. S.; DiMaio, F.; Baek, M.; Baker, D. De Novo Design of Protein Structure and Function with RFdiffusion. *Nature* **2023**, *620* (7976), 1089–1100. https://doi.org/10.1038/s41586-023-06415-8.

(27) Cao, L.; Coventry, B.; Goreshnik, I.; Huang, B.; Sheffler, W.; Park, J. S.; Jude, K. M.; Marković, I.; Kadam, R. U.; Verschueren, K. H. G.; Verstraete, K.; Walsh, S. T. R.; Bennett, N.; Phal, A.; Yang, A.; Kozodoy, L.; DeWitt, M.; Picton, L.; Miller, L.; Strauch, E.-M.; DeBouver, N. D.; Pires, A.; Bera, A. K.; Halabiya, S.; Hammerson, B.; Yang, W.; Bernard, S.; Stewart, L.; Wilson, I. A.; Ruohola-Baker, H.; Schlessinger, J.; Lee, S.; Savvides, S. N.; Garcia, K. C.; Baker, D. Design of Protein-Binding Proteins from the Target Structure Alone. *Nature* **2022**, *605* (7910), 551–560. https://doi.org/10.1038/s41586-022-04654-9.

(37) Wang, J.; Lisanza, S.; Juergens, D.; Tischer, D.; Watson, J. L.; Castro, K. M.; Ragotte, R.; Saragovi, A.; Milles, L. F.; Baek, M.; Anishchenko, I.; Yang, W.; Hicks, D. R.; Expòsit, M.; Schlichthaerle, T.; Chun, J.-H.; Dauparas, J.; Bennett, N.; Wicky, B. I. M.; Muenks, A.; DiMaio, F.; Correia, B.; Ovchinnikov, S.; Baker, D. Scaffolding Protein Functional Sites Using Deep Learning. *Science (1979)* **2022**, *377* (6604), 387–394. https://doi.org/10.1126/science.abn2100.

(50) Junutula, J. R.; Raab, H.; Clark, S.; Bhakta, S.; Leipold, D. D.; Weir, S.; Chen, Y.; Simpson, M.; Tsai, S. P.; Dennis, M. S.; Lu, Y.; Meng, Y. G.; Ng, C.; Yang, J.; Lee, C. C.; Duenas, E.; Gorrell, J.; Katta, V.; Kim, A.; McDorman, K.; Flagella, K.; Venook, R.; Ross, S.; Spencer, S. D.; Lee Wong, W.; Lowman, H. B.; Vandlen, R.; Sliwkowski, M. X.; Scheller, R. H.; Polakis, P.; Mallet, W. Site-Specific Conjugation of a Cytotoxic Drug to an Antibody Improves the Therapeutic Index. *Nat Biotechnol* **2008**, *26* (8), 925–932. https://doi.org/10.1038/nbt.1480.
